# Burkitt Lymphoma—A Guide to Biological Features, Diagnosis and Differential Diagnosis

**DOI:** 10.3390/cancers18040579

**Published:** 2026-02-10

**Authors:** Ioannis Anagnostopoulos, Alberto Zamò, Heike Horn, Annette Staiger, German Ott

**Affiliations:** 1 Institute of Pathology, University of Würzburg, 97080 Würzburg, Germany; alberto.zamo@uni-wuerzburg.de; 2 Department of Clinical Pathology, Robert-Bosch-Hospital, 70376 Stuttgart, Germany; heike.horn@ikp-stuttgart.de (H.H.); annette.staiger@ikp-stuttgart.de (A.S.); german.ott@rbk.de (G.O.); 3 Dr. Margarete Fischer-Bosch-Institute of Clinical Pharmacology, Stuttgart and University of Tübingen, 70376 Stuttgart, Germany

**Keywords:** Burkitt lymphoma, Epstein-Barr virus, *MYC* rearrangement, high-grade B-cell lymphoma, 11q aberration, diffuse large B-cell lymphoma, B-lymphoblastic leukemia/lymphoma

## Abstract

Burkitt lymphoma (BL) is an aggressive mature B-cell lymphoma that represents one of the most studied human malignancies. Initially described in equatorial Africa by the Irish surgeon Denis P. Burkitt, African (endemic) Burkitt lymphoma was the first human neoplasm shown to be associated with a virus, the Epstein–Barr virus (EBV), and also the first human neoplasm shown to harbor a recurrent chromosomal aberration, the t(8;14) (q24;q32) translocation that led to the identification of the central role of the *MYC* gene in tumorigenesis. In this review, we provide a brief historical introduction, followed by a presentation of important aspects of epidemiology, pathogenesis, and of diagnostic features including morphology, cytogenetics and molecular findings. We also provide a comprehensive overview of the findings convincingly demonstrating that subtyping of BL into EBV-positive and EBV-negative better describes the biological heterogeneity of this lymphoma entity than the historical subtyping into endemic, sporadic, and immunodeficiency-associated. As the distinction of BL from other B-cell lymphomas is important for providing optimal oncological care, we also discuss the differential diagnosis and how this lymphoma can be distinguished from other aggressive B-cell lymphomas.

## Simple Summary

Burkitt lymphoma is a rare aggressive cancer of the lymphatic system deriving from mature B lymphocytes. Initially described in 1958 by the Irish surgeon Denis P. Burkitt. It represents the first human cancer shown to be associated with a virus, the Epstein-Barr virus, and a recurring genetic abnormality a chromosomal translocation involving the long arms of the chromosomes 8 and 14. This review presents an overview of the current knowledge regarding world-wide distribution, risk factors, and the known causes for development of this cancer. As Burkitt lymphoma is not only rare, but also shares several features with other aggressive cancers of the lymphatic system, the complex process of arriving at a precise diagnosis is described as well.

## 1. History of Burkitt Lymphoma

**Figure 1:**
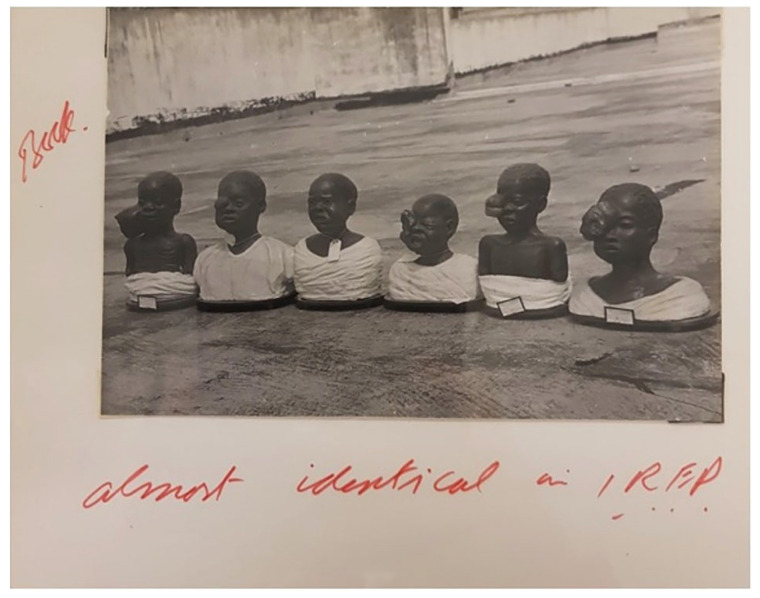
Plaster busts of young children with Burkitt’s lymphoma (WTI/DPB/B/7/4b ‘Album, maps and diagrams relating to cancers in Africa,’ Denis Parsons Burkitt Collection, Wellcome Archive, London, UK). Original figure published in [5].

In 1958, the Irish surgeon Denis P. Burkitt described 38 children in Uganda with rapidly enlarging jaw sarcomas that were uniformly fatal. In that paper, he noted the unique combination of jaw and orbital involvement and a predilection for extranodal spread to the adrenal glands, kidneys, liver, thyroid, pancreas, stomach and ovaries [1]. Shortly thereafter observations by O’Connor and Davies found that the histologic appearance of the facial tumors was identical to that of lymphomas characterized by abdominal masses, and a lymphoid origin was confirmed [2]. It is of interest that earlier references to BL exist either in the form of African wood art, plaster models resembling facial BL (Figure 1), or in clinical notes written as early as 1897 [3]. At a meeting in Paris in 1962 sponsored by the International Union Against Cancer, this lymphoma was designated as Burkitt’s lymphoma [4].

Burkitt was intrigued by the apparent geographic limitation of this tumor. His renowned “tumor safaris” defined a “lymphoma belt” with distinct topographic and climatic features that originally suggested a mosquito-borne etiologic agent. Burkitt and his colleagues at Makerere University in Kampala, Uganda can be credited with alerting the scientific world to the investigative potential of this unusual African lymphoma. In 1961, the young virologist M. Anthony Epstein happened to listen to and became fascinated by Burkitt’s report in London during one of his visits home, and a cooperation was initiated. Epstein started to search for a virus in Burkitt’s tumor specimens using electron microscopy—initially without success. Two years later, Epstein managed, with the help of his colleagues Yvonne M. Barr and Bert G. Achong, to establish several continuously growing cell lines from BL tumor specimens [6]. Finally in 1964, Epstein and colleagues were able to identify by electron microscopy, in a small fraction of cells in one of these cell lines, the presence of intracellular particles that had the characteristic morphological features of a virus [7]. The virus was further characterized and named eponymous Epstein–Barr virus (EBV) by Werner and Gertrude Henle [8] and became the first virus implicated as a causative factor of a human cancer. Further studies gradually recognized that cases of BL also occur outside Africa, including high-income nations [9,10]. Characteristic abnormalities involving extra bands on the long arm of chromosome 14 with corresponding loss of material in chromosome 8 were identified in BL in 1972 [11]. This observation led to the discovery of reciprocal translocations that juxtaposed the *MYC* oncogene on chromosome 8 and immunoglobulin loci on chromosomes 2, 14, or 22, resulting in deregulated MYC expression and uncontrolled tumor proliferation [12]. The geographic distribution, initially observed by Burkitt, also correlated with regions that are holoendemic for *Plasmodium falciparum* (*P. falciparum*) malaria, and areas with successful malaria eradication programs did not report this tumor [13].

## 2. Epidemiology

### 2.1. Geographical Patterns of Incidence

The age-standardized incidence rates (ASRs) of BL vary 3-fold to 5-fold within and across continents. The highest ASRs are observed in equatorial Africa (>4 cases per million person-years) where BL is considered endemic in a geographical belt spanning 10° N to 10° S of the equator. BL rates are highest in regions (such as Malawi, Uganda and Cameroon) with high temperature suitability for transmission of *P. falciparum*, a unicellular protozoan parasite that can cause malaria in humans, and low in areas with low temperature suitability for *P. falciparum* transmission (Ethiopia, around Nairobi in Kenya, and Mali). BL ASRs in tropical regions of Central and South America are intermediate (2–3.9 cases per million person-years) or low (<2 cases per million person-years) and vary 3-fold across countries. BL ASRs in North America and Europe are in the intermediate range, but geographically variable, with some countries like Canada, Czech Republic, Belarus, Ukraine, and Poland being in the low range. The lowest BL ASRs have been recorded in Asia, particularly in India and China, where rates are one-sixth of that in USA and one-twentieth of that in Uganda. This fact is surprising as the epidemiology of the best-known risk factors for BL (*P. falciparum* and EBV) is more similar between Africa and China than between the USA and Europe. These differences could be due to the different pathogenicity of EBV strains for BL between Asia and Africa. Interestingly, African EBV strains are more closely related to those circulating in USA and Europe [14] than to those circulating in Asia. The prevalence of EBV association in BL is approximately 95% in sub-Saharan Africa [15], approximately 50% in Brazil [16] and approximately 20% in Europe and USA [17], while its prevalence in Asia is unknown.

### 2.2. Sex and Age Patterns

BL rates are two-fold to four-fold higher in males than in females in every country and show geographic variations in both high-income countries and low-income countries. The male predominance is a consistent feature of all BL in all age groups and also across geographical regions. Possible causes for this phenomenon might be due to a role of BL-associated recessive genetic factors on sex chromosomes [18,19] or other host or environmental factors such as hormonal or microbial sex differences [20].

BL can occur at any age, but the proportion of BL diagnosed in the age group 0–14 years compared with all age groups is approximately 13% in Europe and USA, approximately 50% in Israel and Turkey, and 62–100% in some African regions, possibly because BL in adults is missed in those countries. A trimodal pattern occurs in the USA, with an early peak in the pediatric age group (0–18 years), a second peak in the group around 40 years of age, and a third peak in the older age group around 70 years of age [21]. Fifty percent of all BL cases in the USA occur in adults aged 20–59 years. Although the data are sparse for ages > 35 years in Africa, a bimodal age pattern emerges when data are grouped by region, except for Southern Africa where the pattern is more like that in the USA, Australia and the UK. The observed heterogeneity in BL may reflect epidemiological associations with primary and reactivated EBV infection rates. Several studies have shown that the prevalence of EBV infection in children varies from 10 to 90%, depending on age, geographic location, and race/ethnicity even in the same area [22,23,24,25]. Additional data have shown that children may acquire primary EBV infection at later age in developed countries. In particular, studies from USA, England, Wales and Japan have revealed a decrease in primary infection, especially among young children [26,27,28]. The explanation for this might be an improvement in socioeconomic status, as higher household income and education level were significantly associated with a lower prevalence of EBV antibody in one study [23]. A study performed on a large cohort in China showed that EBV-DNA positivity gradually increased after age of 40. The median age of patients with primary infection was 22 years while the median age of those with reactivation was 51 years [29]. This is in line with the results of one study from Germany demonstrating that the proportion of EBV-positive patients increases with age at sporadic BL diagnosis [19].

### 2.3. Variants

There are three recognized BL variants based on the epidemiology of this malignancy: endemic, sporadic, and immunodeficiency-associated. BL morphology and immunophenotype is identical across all variants; however, there are significant differences in epidemiology and clinical features. The most common one, corresponding to the variant initially described by Burkitt, is the endemic form of BL. Endemic BL (eBL) is a pediatric malignancy with peak incidence between 6 and 8 years of age and a predominance in males. eBL occurs in malaria-holoendemic areas of Africa and Papua New Guinea [30,31]. Historically, eBL involved the jaw, the orbit, or both, but contemporary studies show that abdominal involvement is now more common [32]. It is postulated that jaw involvement is related to poor dentition, thus allowing EBV to gain access to jaw marrow cells, and that the change in the clinical presentation is likely related to improved dentition. Bone marrow may be involved, but frank leukemia is uncommon. CNS involvement occurs in less than 10% of cases manifesting as cranial nerve palsies or spinal cord compression. eBL is associated with EBV in approx. 95% of the cases and elevated titers of EBV are associated with an increased risk [33].

In contrast to eBL, cases of sporadic BL (sBL) occur throughout the world. The median age at presentation is 10 years, with additional peaks at 40 years and 75 years of age [21]. sBL represents approx. 50% of childhood lymphomas, but less than 3% of all lymphomas in USA and Western Europe. Patients older than 60 years represent only 20% of the cases, but the incidence in this population is rising [21]. sBL, like eBL, also occurs more frequently in males with no racial or ethnic predisposition. sBL can affect any organ but often manifests as a rapidly enlarging abdominal mass, with involvement of the ileocecal region causing bowel obstruction. Involvement of both breasts is uncommon and can mimic inflammatory breast cancer. CNS involvement occurs in up to 20% of the cases and bone marrow is involved in 20–30% of cases. In contrast to eBL, EBV is detected in 10–30% of the cases, most often in patients over the age of 50 years [34].

The third subtype of BL emerged with the human immunodeficiency virus (HIV) epidemic. The median age of immunodeficiency-associated BL (ID-BL) is 40–45 years, and cases are equally distributed between the sexes. ID-BL most commonly arises in patients infected with HIV and was the first described HIV-associated lymphoma [35]; it is currently considered an AIDS-defining condition. There are also a few case reports of ID-BL in post-transplant patients, but BL is not common in this setting [36]. HIV-associated BL occurs early in HIV infection and before CD4+ T-cell numbers drop [37]. The incidence of HIV-associated BL has remained stable in the USA since the introduction of combination antiretroviral therapy for HIV. The clinical manifestation of HIV-associated BL often involves the gastrointestinal tract and bone marrow, while CNS involvement occurs in 20–30% of the cases. ID-BL is associated with EBV infection in 20–40% of the cases; the frequency of EBV-positive cases has, however, been reported to be as high as 71% in a series from Brazil [38].

A different epidemiologic pattern of BL has been identified in South America, where EBV is detected in approximately half of the cases of all ages. Some studies have also highlighted the fact that BL occurring in children less than 5 years of age is consistently associated with the EBV infection [16]. This finding is in contrast to eBL where no age segregation of EBV positivity in children has been identified. Also, the prevalence of EBV in BL is not homogeneous in all regions of Brazil, with regions in the north showing a higher frequency of EBV association in BL.

As the global population becomes increasingly mixed, this traditional epidemiologic subclassification of BL may no longer be practical. For instance, sBL can also occur in Africa, accounting for some of the rare EBV-negative cases reported from this continent, and ID-BL can occur in the same regions in Africa as eBL. Dennis Wright, one of the first pathologists analyzing BL, raised this question in a letter foreseeing that EBV-positive BL is different from EBV-negative BL in cellular biology and pathogenesis [39]. Several publications, analyzing the molecular pathogenesis of BL more deeply, reported that EBV status defines specific BL phenotypes (see below). Consequently, the current 5th edition of the WHO classification of Haematolymphoid Tumours (WHO-HAEM5) recommends subtyping of BL based on EBV status rather than epidemiology [40].

### 2.4. Risk Factors

The best-characterized risk factors for BL in endemic settings are recurrent infection with *P. falciparum* prior to infection with EBV during childhood [41]. The occurrence of BL is correlated with intensity of malaria transmission [13] and infection with multiple parasite clones [42]. BL risk is decreased in children carrying genetic variants that protect against malaria, such as the sickle cell trait [43], suggesting that children lacking protection against *P. falciparum* infection are particularly vulnerable to BL development. A recently performed genome-wide association study identified an African specific locus on chromosome 21q22.12, near *RUNX1* tagged by the rs111457485 allele, associated with reduced BL risk and also with better survival in patients with abdominal-only BL in exploratory analyses [44].

Germline factors may contribute to rare familial cases, such as the reported germline variants in *TCF4* and *CHD8* reported in two related children in Uganda [45]. Risk factors reported in the EBV-positive sBL setting include inborn errors of immunity, such as Purtilo–Duncan syndrome/X-linked lymphoproliferative disease (OMIM 308240) or XMEN disease (MIM 300853) and DNA repair disorders, such as Ataxia telangiectasia (MIM 615919), and developmental disorders, such as Williams–Beuren syndrome (MIM 194050).

Interestingly, also nutritional deficiencies as low intake of dietary selenium [46] and magnesium have been implicated as risk factors for BL. The identification of XMEN disease, a genetic disorder of the magnesium ion transporter that is associated with intracellular magnesium deficiency in natural killer cells and selective deficits in EBV immunity, led to the discovery that intracellular magnesium deficiency is a potential cofactor in EBV control. In a study of African children with and without BL, plasma magnesium levels were found to be significantly lower in children with BL than in healthy children. This study also showed that healthy women, who did not have XMEN but a high EBV load, more likely exhibited a magnesium deficiency than women with a low EBV load [47]. This suggests that dietary magnesium deficiency might impair EBV control.

As previously stated, individuals with immunosuppression resulting from HIV infection or receiving immunosuppressive therapy to prevent transplant rejection, and individuals with inborn errors of immunity, exhibit a considerably increased risk of BL. Populations with a HIV infection in the USA, Europe and Australia have a 50–60-fold higher risk of developing BL than the general population [48]. In contrast, the increase of risk of BL in HIV-infected individuals living in Africa is a modest two-fold to six-fold [49]. The risk of developing BL in HIV-infected individuals in India and China also seems to be substantially lower than that reported in high-income regions [50]. The underlying reasons are unclear, but might include under-detection, diagnostic misclassification and competing mortality.

## 3. Pathogenesis

### 3.1. MYC Rearrangement

Although not specific for BL, *MYC* rearrangement is the primary molecular event in nearly all cases [40,51]. This rearrangement leads to activation of MYC through juxtaposition of the transcriptional enhancer element of one of the immunoglobulin gene loci with *MYC*. The t(8;14)(q24;q32)/*I*GH*::MYC*, located on the derivative chromosome 14, is present in roughly 80% of patients with BL. Less frequently, in about 15–20% of BL cases, *MYC* is juxtaposed to the IGK or IGL locus via the t(2;8)(p12;q24)/IGK*::MYC* or t(8;22)(q24;q11)/IGL*::MYC*, respectively. These less frequent abnormalities are known as variant translocations and are located on the derivative chromosome 8. The two main mechanisms resulting in IG*::MYC* in BL are aberrant class switch recombination (CSR) and aberrant somatic hypermutation (SHM), which both take place in the germinal center [40,52]. Aberrant CSR results in location of the IGH*::MYC* breakpoints in the switch regions of the constant-gene segments encoding the immunoglobulin isotypes IgM, IgD, IgG, IgA and IgE. In contrast, breakpoints derived from aberrant SHM are located within or adjacent to rearranged V(D)J genes. Under normal physiologic conditions, CSR and SHM of immunoglobulin genes, key features in the generation of the adaptive humoral immunity, are mediated by enzyme activation-induced cytidine deaminase (AID) (also called AICDA). AID induces point mutations by deaminating cytidine to uracil, and because of this it is easy to identify mutations by recognizing its specific genetic signature. AID also induces double-strand DNA breaks and triggers apoptosis [51,53].

The breakpoints in the chromosome 14 IGH region vary; most breakpoints in eBL are located within or adjacent to rearranged V(D)J genes (suggesting SHM as a translocation mechanism), whereas in sBL and ID-BL, the breakpoints occur within the IGH switch or constant regions (suggesting class switch recombination as a supposed translocation mechanism) [52,54]. In the variant translocations, the breakpoints on chromosomes 2 and 22 are frequently located 5′ of the IGK and IGL constant region segments [55]. Breakpoints of *MYC* in the IGH*::MYC* also vary and fall into three classes: those located within the *MYC* locus affecting the first exon or the first intron are class I breakpoints, those immediately upstream (5′centromeric) of MYC are class II breakpoints, and those located far upstream (5′centromeric) are class III breakpoints. In sBL and ID-BL, class I and II seem to be predominant, whereas class III breakpoints, which can map over hundreds of kilobases upstream from the *MYC* basal promoter, are enriched in eBL. In cases with variant translocations, the breakpoints are typically located downstream (3′) of *MYC*, even up to 2 Mb [40,52]. This variation might impact the sensitivity of commonly used FISH tests.

The molecular consequence of all three translocations is deregulated expression of the *MYC* oncogene via enhancer hijacking. This takes place on the derivative chromosome 14 for IGH*::MYC* and on the derivative chromosome 8 for IGK*::MYC* and IGL*::MYC*, resulting in consecutive expression of MYC, which contributes to the neoplastic process by upregulating cell proliferation, loss of apoptotic control and tumor progression. In some patients, cryptic insertions of *MYC* into an immunoglobulin locus or of immunoglobulin enhancer elements into the *MYC* locus occur instead of typical chromosomal translocation, which might be hard to detect using conventional diagnostic tests as fluorescence in situ hybridization (FISH) or conventional cytogenetics [56,57]. These deceptively *MYC*-negative BL generally present with a typical BL immunophenotype and a similar gene and mRNA expression profile as those seen in *MYC*-rearranged BL cases (see below). Leucci et al. found that hsa-mir-34b physiologically targets and inhibits MYC expression, and that has-mir-34b is downregulated in *MYC* rearrangement-negative BL cases, a finding that might explain MYC overexpression in these cases [57]. These findings support the idea that alternative pathogenetic mechanisms, such as miRNA dysregulation, might occur in a small subset of BL cases.

### 3.2. Genomic and Molecular Alterations

Mouse models have confirmed that *MYC* translocation alone is insufficient to drive BL lymphomagenesis [58,59,60]. As a matter of fact, MYC overexpression induces apoptosis in non-malignant B cells [61]. Therefore, additional pathogenetic changes are required, either before or within a short window after MYC activation to lead to full BL lymphomagenesis. Recent molecular studies have identified many recurrent genetic abnormalities in addition to *MYC* translocation. The commonly affected genes and pathways include (a) B-cell receptor (BCR)/phosphoinositide 3-kinase (PI3K) signaling (e.g., *ID3*, *TCF3*, *FOXO1*, *MIR17HG* and *PTEN*); (b) proliferation and survival (e.g., *MYC*, *CCND3*, *CDKN2A*, *TP53*, *BCL2L11*, *RFX7*, *USP7*, *SIN3A* and *TFAP4*); (c) G protein-coupled receptor/-sphingosine-1phosphate (S1P) signaling (e.g., *P2RY8*, *RHOA*, *GNA 12* and *GNA13*); (d) SWItch/Sucrose Non-Fermentable (SWI/SNF) complex (i.e., *SMARCA4*, *ARID1A*, *BCL7A*, *CHD4* and *BCL11B*); and (e) epigenetic regulation (e.g., *KMT2A*, *HIST1HE* and *CHD8*) and others (e.g., *PCBP1*, *TFAP4*, *FBXO11*, *DDX3X*, and *ETS1*).

Highly recurrent alterations involving the transcription factor TCF3 and its negative regulator ID3 are essentially absent from other *MYC*-negative germinal center-derived B-cell lymphomas. *TCF3*-activating mutations are found in 10–20% of BL cases, whereas inactivating mutations in *ID3* have been identified in 50–60% of BL cases [62,63]. Thus, these findings indicate an important role for BCR signaling in BL pathogenesis. Functional analyses have suggested that mutations in *ID3* and/or *TCF3* enhance *TCF3* transcriptional activity, thereby allowing TCF3 to transactivate the genes encoding immunoglobulin heavy and light chains as well as the BCR signaling subunits CD79A and CD79B [62]. In addition, TCF3 directly represses *PTPN6*, which encodes SHP-1, a negative regulator of BCR signaling. Thus, the constitutive activation of TCF3 promotes tonic BCR signaling and facilitates the PI3K signaling pathway. Mouse models have shown that expression of activated *MYC* combined with *PI3K* pathway activation in germinal center B cells leads to tumors that closely resemble human BL [64].

In addition to its genomic translocations, *MYC* is the most commonly altered gene in BL with mutations occurring in 70% of BL cases. The mutations mainly affect the transactivation domain and are enriched in target motifs of the SHM machinery [65]. These alterations may drive uncontrolled tumor proliferation and activate BCR signaling, which engages the PI3K pathway and promotes cell survival. *MYC* mutations are also clustered around phosphorylation sites which can prevent the degradation of MYC and enhance its stability [55]. Also, mutations in *CCND3* and its negative regulator *CDKN2A* contribute to cell proliferation and survival [65,66]. Activating mutations in *CCND3* and inactivating mutations in the tumor suppressor gene *TP53* have been identified in 40% and 50% of BL, respectively [62]. The presence of mutations deregulating both genes can explain the high proliferation rate of tumor cells in BL as TP53 inactivation and altered expression lead to increased proliferation and evasion of apoptosis.

Early studies showed that *TP53* mutations in BL inactivate the p53 tumor suppressor and these alterations may be enriched in cases that are refractory to chemotherapy. Loss of TP53 promotes MYC-induced transformation by blocking the apoptosis that results when the MYC is overexpressed in B cells. In addition to *TP53* mutations, the tumor suppressors *ARF* and *USP7* are inactivated in BL, therefore augmenting MDM2-mediated degradation of TP53 [67]. The tumor suppressor *DDX3X* is also frequently mutated or deleted in BL, which lowers MYC-induced proteotoxic stress at disease initiation [62,65,68]. Later, the malignant cells increase expression of the Y chromosome-encoded paragogue DX3Y, thus restoring full protein synthesis, which may account in part for the higher prevalence of BL among males [68]. Another MYC regulator that is inactivated in BL is SIN3A, which deacetylates MYC and decreases its activity as a transcriptional activator [69].

Sphingosine-1-phosphate signaling is deregulated in BL as a result of mutations and altered DNA methylation. Inactivation of the G-protein-coupled receptor P2RY8 and its downstream signaling mediator Galpha13 (*GNA13*) alters the mobility and dissemination of BL cells [70].

The SWI/SNF chromatin remodeling complex also plays an important role in BL pathogenesis. Mutations in *SMARCA4* and *ARID1A* are common in this complex and are observed in a mutually exclusive pattern. *SMARCA4* mutations are present in approximately 40% of BL cases and target the helicase domain, while truncating *ARID1A* mutations have been described in 20–30% of BL cases. Binding of helicase-deficient mutated SMARCA4 to its target genes is associated with demethylation of its binding sites and lack of target gene expression due to absence of helicase activity. Further genomic mutations of other members of the SWI-SNF complex (*BCL7A*, *CHD4*, *BCL11B*) have been also identified in BL; they are however uncommon.

### 3.3. The Role of EBV

Despite 60 years passing since the discovery of EBV in BL tumor cells, there are still many unresolved questions and paradoxical observations as to how this virus contributes to BL pathogenesis. One of the paradoxes is that while EBV is widespread globally and persists in latent form in a large portion of the population, only a small fraction of these individuals develops BL. This discordance indicates that EBV infection alone does not fully induce BL development.

To understand the role of EBV in the pathogenesis of BL, it is worth focusing on what has been learnt from studies in eBL children. In a seminal study in Uganda, antibodies to EBV viral capsid antigen were significantly increased prior to the emergence of BL, thus providing evidence of the causal role of EBV [71]. Later studies showed elevated antibodies to EBV lytic antigens in patients with BL but not in healthy individuals [72]. As already stated, the EBV genome is detected in most patients with eBL. Subsequent analysis showed that the viral genome is clonal, indicating that EBV infection preceded the malignant transformation event [73]. Loss of the EBV genome from BL cell lines usually results in apoptotic cell death [74].

Another paradox of determining EBV’s role in BL pathogenesis is that although EBV encodes several latent proteins essential for B-cell immortalization, only one latent protein, EBNA-1, is consistently expressed in BL [75]. Inhibition of EBNA-1 in BL cell lines results in apoptosis [76]. Consistent with this finding, Holowaty showed that EBNA-1 binds to a deubiquitinase (USP7) [77]. USP7 binds to TP53 and MDM2, stabilizing the proteins. Binding of EBNA-1 to USP7 destabilizes TP53, potentially suspending apoptotic responses. It is of interest that *USP7* is mutated in EBV-positive BL [65]. In addition to EBNA-1, the RNA-pol III non-translated RNAs termed EBV-encoded small RNAs (EBER)-1 and EBER-2 are also consistently expressed in EBV-positive BL, as well as in all latently infected cells. Because of their high level of expression, the EBERs are easily detected by chromogenic in situ hybridization and are thus useful for routinely identifying EBV-infected cells in tissue specimens. Their functional role in BL remains controversial. Takada et al. used the EBV+ BL line Akata, which over time lost the EBV episome. They were able to show that reintroduction of EBNA-1 restored resistance to apoptosis via induction of BCL-2 [78]. A variant of the promoter for the immediate early protein, Zta, a protein required to initiate the lytic EBV cycle, has been found more frequently in patients with BL than in healthy control individuals. This observation points to a possible role for lytic reactivation of EBV in BL pathogenesis [79] In this context, it is of interest that recent experimental data have shown that the EBV lytic cycle induces *MYC*-IGH proximity in B cells and predisposes to the appearance of the t(8;14) translocation [80].

EBV-encoded BART microRNAs (miRNAs) are also detected in EBV-positive BL. Their presence was associated with a different transcriptional profile of BLs, pointing to a possible oncogenic role [81]. Forced loss of the EBV episome followed by expression of BART miRNAs rescues the cells from apoptosis via inactivation of CASP3 [82]. Another piece of evidence of an important role for miRNAs in BL comes from a study that demonstrated the presence of EBV miRNA in EBER-negative BL cells [83]. These data provide some explanation of how EBV-negative BL can emerge from an EBV-infected *MYC*-translocated cell.

A consensus has emerged that the role of EBV in BL pathogenesis involves its impact on disarming apoptotic pathways in B cells, contributing to their malignant transformation. To evade apoptosis, B cells must tolerate and survive constitutive *MYC* activation, a fact that may be attributable to EBV [84]. The pathogenic effects of EBV in B cells likely precede *MYC* rearrangement [73], suggesting that EBV infection may play an early role in the oncogenesis of BL.

A number of research groups have addressed the possibility of whether there is a particular strain or variant of EBV associated with BL that had more oncogenic potential. However, the results have been not consistent and the question remains open. EBV strains are subclassified into type 1 and 2 based on differences in sequence of the genes that encode EBV nuclear antigens [85]. Type 1 is more prevalent in patients with eBL, whereas type 2 EBV is more commonly found in females than males with EBV and is associated with decreased expression of key genes in the immunoproteasome complex when compared with type 1 EBV [86,87]. However more studies are needed to confirm these observations.

For the EBV-negative BL subtype, a hit-and-run mechanism has been proposed where EBV plays an initiating role in oncogenesis but the viral episome is lost [88]. Interestingly, two studies detected EBV miRNA but not EBER in BL samples [83,89]. This raises the intriguing question of whether EBV-negative BL are actually derived from EBV-infected B cells. Analysis of mutations in EBV-negative BL has further supported the hypothesis that EBV can be lost as compensatory mutations have occurred that substitute for the functions of EBV-encoded proteins [88]. Other studies have, however, identified unique gene expression profiles between EBV-positive and EBV-negative BL [90] and evidence of ongoing somatic hypermutation only in EBV-positive BL [65], which argue for different pathogenetic mechanisms behind EBV-positive and EBV-negative BL.

### 3.4. Activation-Induced Cytidine Deaminase (AID) and BL

When the *MYC* translocation was first identified, there was speculation that it was mediated by the VDJ B cell recombinase. However, Robbiani et al. provided in 2008 evidence that the enzyme AID (also called AICDA), was actually the cause of the *MYC* translocation [91]. As already stated above, AID has a role in antibody diversification through SHM and CSR. Because of the potential danger to the cell of having an enzyme capable of modifying the genome, AID expression and function are tightly regulated at multiple levels including transcriptional, cellular localization, and nuclear half-life [92,93,94].

In a pathological context, AID facilitates mutations and rearrangements in non-IG genes such as *MYC*, *PAX5* and *DDX6*, which are all linked to lymphomagenesis [95], and increased AID expression has been reported in several lymphomas including diffuse large B-cell lymphoma [96], follicular lymphoma [97], chronic lymphocytic leukemia [98], and BL [65].

While overexpression of AID has been observed in BL, the causes of this phenomenon are still unclear. As presented in the previous sections, both EBV and *P. falciparum malaria* are known cofactors in the etiology of BL. However, how these two pathogens drive BL pathogenesis is not yet understood. There are several lines of evidence suggesting that EBV is a potential driver of increased AID activity: (i) higher AID mRNA levels have been reported in peripheral blood and tonsils of children with high EBV load [99]; and (ii) gene expression analysis of BL revealed significantly higher expression of *AICDA*, the gene that codes for AID protein, and its associated mutations, in EBV-positive than in EBV-negative BL [65]. The EBV-encoded nuclear antigen EBNA-3C and latent membrane protein LMP-1 have been shown to induce AID expression in B cells [100,101]. However, the role of the EBV-encoded nuclear antigen EBNA-2 remains unclear: it has been shown that EBNA2 suppresses AID expression in proliferating cells [102], but in contrast, lymphoblastoid cell lines characterized by expression of all EBV latent genes still express AID.

Several possible mechanisms have been also suggested by which *P. falciparum* could increase the risk of BL. Some of these mechanisms include suppression of EBV-specific cytotoxic T-cell responses, increased EBV reactivation and increased germinal center transition of EBV-infected cells [41,103]. *Plasmodium* infection alone can induce aberrant AID expression in B cells outside of the germinal centers in a mouse model [104]. In another study, repeated infection of TP53 deficient mice with *P. chabaudi* induced lymphomas that had the characteristic *MYC* translocation of the IgH. Using then an AID knockout mouse, the authors could show that the capacity to induce a translocation was dependent on AID [105]. *P. falciparum* DNA is a ligand for toll-like receptor 9 that, following stimulation, induced AID expression in B cells [106,107]. The B-cell cytokine BAFF is induced during *P. falciparum* infection and has been shown to increase AID activity in B cells [108]. A more recent study showed that sustained and aberrant activation of AID, induced by both direct and indirect effectors of *P. falciparum*, is a key mechanism in the etiology of eBL. This study also provided evidence that *P. falciparum* and EBV synergized to induce sustained expression of AID in B cells, thus potentially influencing the *MYC* translocation characteristic of BL [109].

If *P. falciparum malaria* and EBV act in concert to drive eBL, what might be the factors that drive the other variants of BL? One possibility is that factors that induce chronic activation of B cells and aberrant AID expression could trigger the *MYC* translocation. This model relies on BCR stimulation through extrinsic activation. Such evidence is present [110,111] and suggests that other chronic infections, including HIV, could trigger lymphomagenesis [90,112]. However, analysis of BCR signaling in sBL is more indicative of mutations allowing tonic BCR signaling and is not necessarily antigen selected [62,110]. Due to the relatively low incidence of sBL, understanding of its etiology remains challenging.

In summary, with the key molecular event in BL being *MYC* translocation with an IG gene partner, this neoplasm is a multifactorial disease influenced by various factors, including germline predisposition, infection, genomic and epigenomic alterations in specific cellular pathways. These factors eventually drive the full transformation of the rearranged MYC clone, leading to uncontrolled proliferation of lymphoma cells.

### 3.5. Epstein-Barr Virus Presence or Absence as a Defining Feature

As stated above, there is sufficient evidence that EBV-positive BL exhibits molecular differences from EBV-negative BL. To better understand this phenomenon, it is useful to look at the reported differences in studies that compared eBL (almost always EBV-positive) and sBL (almost always EBV-negative).

The first key molecular difference in eBL vs. sBL regards the breakpoint differences in the *MYC* translocation reflecting differences in their timing: in EBV-positive BL, the translocation occurs during SHM, while in EBV-negative BL, it occurs more typically during CSR [113,114,115]. Bellan and colleagues found that EBV-positive BL, regardless of epidemiologic origin, had in its rearranged immunoglobulin heavy chain more somatic mutations and evidence for antigen selection than EBV-negative BL [116]. A subsequent study using next-generation sequencing of IG genes supported the model of an antigen-driven selection of BCRs in eBL [111]. A comparison of the eBL mutational landscape with published data on sBL revealed an almost mutual exclusivity between EBV presence and mutations in *TCF3* and its negative regulator *ID3*, both well-known driver genes in sBL. In this study, a hierarchical clustering of both eBL and sBL cases on *TCF3* target genes, previously reported by Schmitz and colleagues [62], showed that the samples could be classified into EBV-positive and EBV-negative independently of the specific subtype with an accuracy rate of 96% [117]. Grande and colleagues further supported in their study the concept that EBV-positive BL cases harbor fewer driver mutations than EBV-negative BL, especially in genes with roles in apoptosis despite having a higher load of aberrant SHM [65]. Additional studies showed that EBV-negative BL tends to harbor canonical mutations affecting *TCF3*, *ID3* and *CCND3* that are more common in pediatric than in adult cases, while EBV-positive BL tends to be enriched in mutations affecting *DDX3X*, *GNA13* and *FOXO1* [19,118]. The mutations present in EBV-negative BL cases are quite unique to BL, while mutations found in other germinal center-derived aggressive B-cell lymphomas, such as those affecting genes encoding chromatin modifiers, are not seen.

A recent study of the epigenetic landscape of 96 BL cases from different geographic origins demonstrated that the genome-wide DNA methylation-based clustering of BL is primary driven by the association with an EBV infection rather than the epidemiologic variants [119]. In particular, DNA methylation analyses clustered the BL cases into four subgroups: two containing mostly EBV-positive cases and two containing mostly EBV-negative cases. The subgroups enriched with EBV-positive cases displayed increased DNA methylation accompanied by increased epigenetic age and proliferation history compared to the subgroups enriched with EBV-negative cases. Interestingly, the extensive DNA hypermethylation in EBV-positive BL cases, compared to EBV-negative cases, covered regulatory regions of genes frequently mutated in BL, such as *CCND3*, *GNA13*, *TP53* and *USP7,* fitting to the hypothesis that DNA hypermethylation may compensate for the lower frequency of mutations in driver genes observed in EBV-positive BL cases.

### 3.6. Immunogenetic Features of BL

Despite losing the availability of one allele due to the presence of the *MYC* translocation, BL maintains a functional B-cell receptor complex, pointing to the importance of a functional immunological synapse for the survival and proliferation of BL cells. The interest in the immunogenetic features of BL date back to the 1990s, when several papers investigated the genetic features of rearranged immunoglobulin genes in eBL [120], sBL [121] or both [121,122], although in a limited number of cases. These early studies highlighted the preferential use of certain VH families (VH1, VH3 and VH4) as well as the presence of SHM of the rearranged immunoglobulin genes, although with a level of mutation that was lower than that observed in follicular lymphoma. Analyzing the pattern of mutation of the VH4 family genes, Chapman et al. found no evidence for an antigen selection [123]. Later studies confirmed and refined the information regarding a biased VH gene usage, with a very high percentage of cases showing a preference for VH4-34, VH3-30, VH3-21, VH3-07 and VH4-59 [65,124]. The level of SHM was also found to be significantly different between sBL, eBL and AIDS-related BL, with the latter two showing a significant higher percentage of variant nucleotides [116]; the same authors observed also a significantly higher SHM in EBV-positive cases. Interestingly, immunoglobulin light chains also display signs of SHM [122,123], especially in endemic cases [122]. At variance with previous findings regarding heavy-chain genes, the pattern of mutation shows a role for antigen selection [123]. One previously cited high-throughput sequencing study also noted a marked bias for the use of the IGKV3-20 gene segment [65]. It is therefore tempting to speculate about the role of specific antigen stimulation in BL; one interesting study screened BL cell lines for their BCR specificity against an array of allo- as well as auto-antigens, and found two cell lines with reactivity against auto-antigens, namely a SUMOylated isoform of Bystin and an atypically acetylated isoform of HSP40 [125]. Interestingly, the VH4-34 heavy-chain gene also determines an auto-reactivity against erythrocyte antigens, so more research to elucidate the role of autoimmunity in the different forms of BL is warranted.

## 4. Diagnostic Features

To establish a preliminary diagnosis of BL, material from a minimally invasive diagnostic procedure may be sufficient. It is however important to point out that a fine-needle biopsy may not yield sufficient material for all tests required to establish a reliable diagnosis and to distinguish BL from diffuse large B-cell lymphoma and other high-grade B-cell lymphomas that may carry a *MYC* translocation.

### 4.1. Morphology

**Figure 2:**
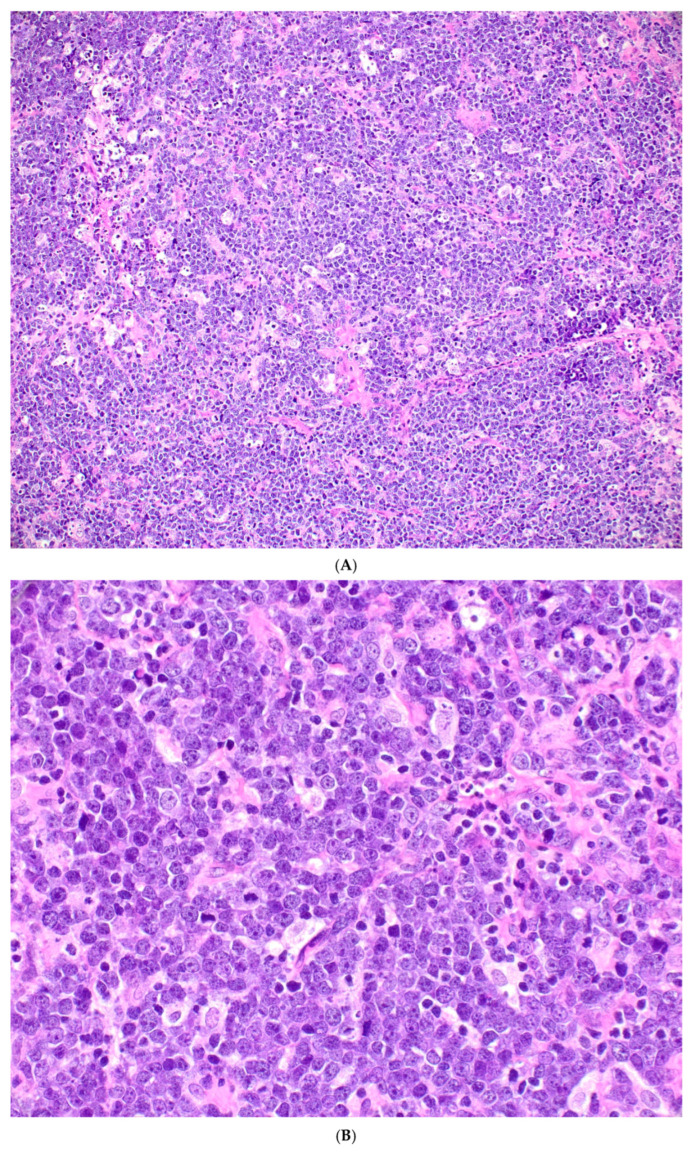

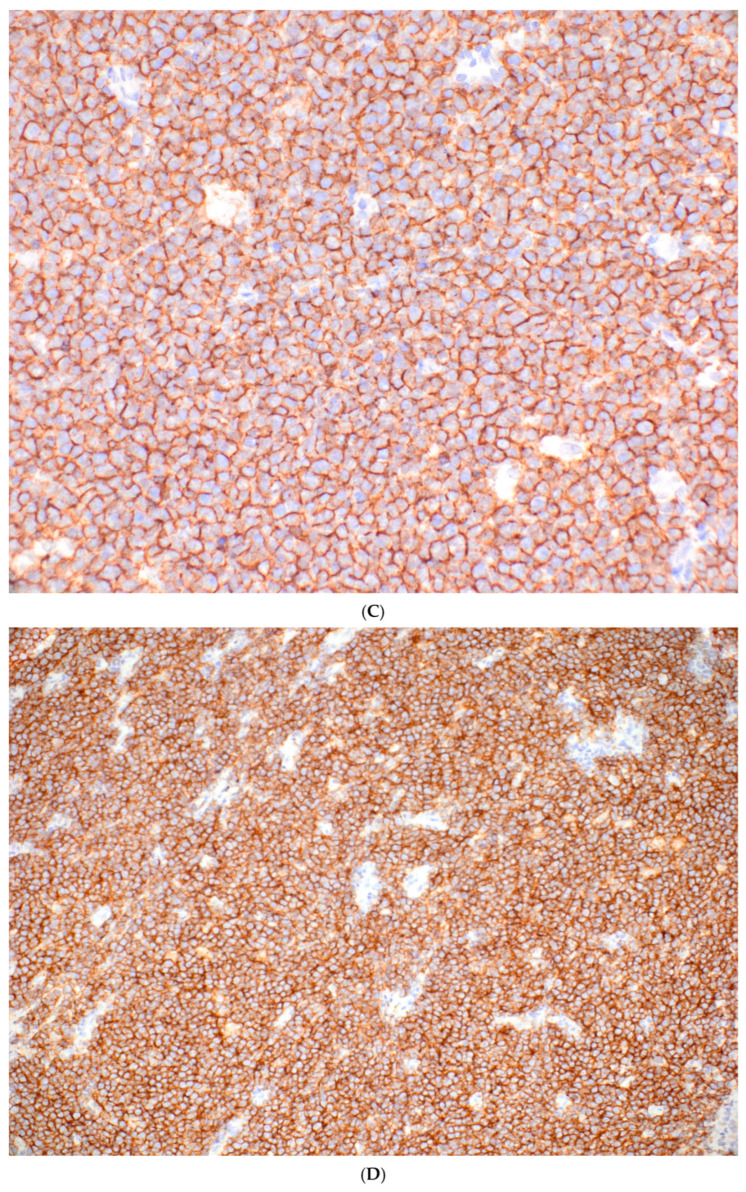

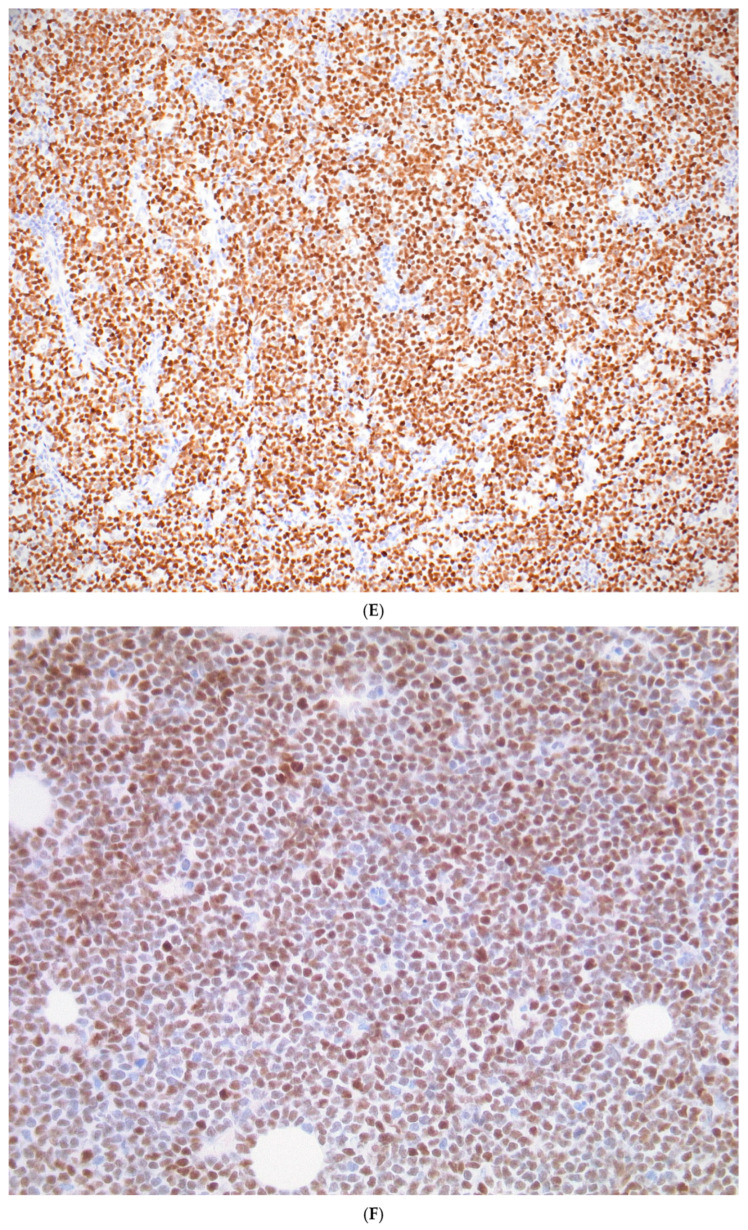

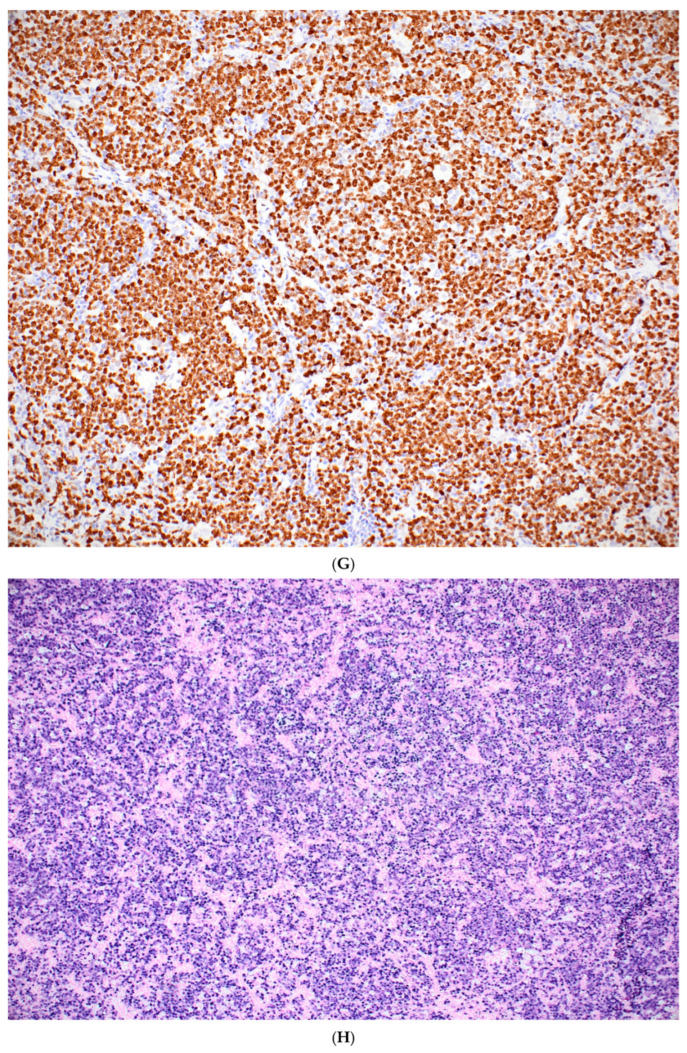

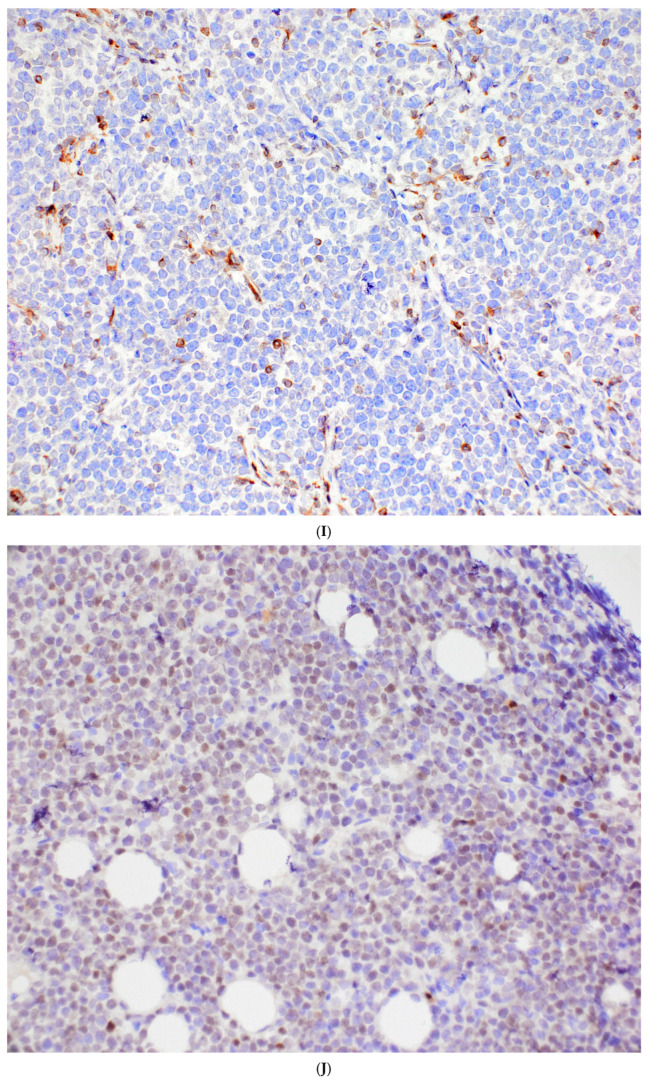
Representative morphology and immunophenotype of BL. (**A**) Low-power view shows a diffuse population of monomorphic lymphoid cells with a starry sky appearance. (**B**) High-power view highlights neoplastic cells that are of intermediate size comparable to the histiocytes. They exhibit round central nuclei harboring several small and distinct nucleoli surrounded by a small rim of cytoplasm. Mitotic figures and apoptotic bodies are present. Immunohistochemical analysis shows that the lymphoma cells express CD20 (**C**), CD10 (**D**), BCL6 (**E**) and MYC (**F**). Ki67 demonstrates a high proliferation rate, close to 100% (**G**). In cases associated with an EBV infection, all neoplastic cells are positive for EBV-encoded small RNA (EBER) (**H**). BCL2 is not expressed by the neoplastic cells (**I**), while IRF4/MUM1 can be expressed with low intensity (**J**).

**Figure 3:**
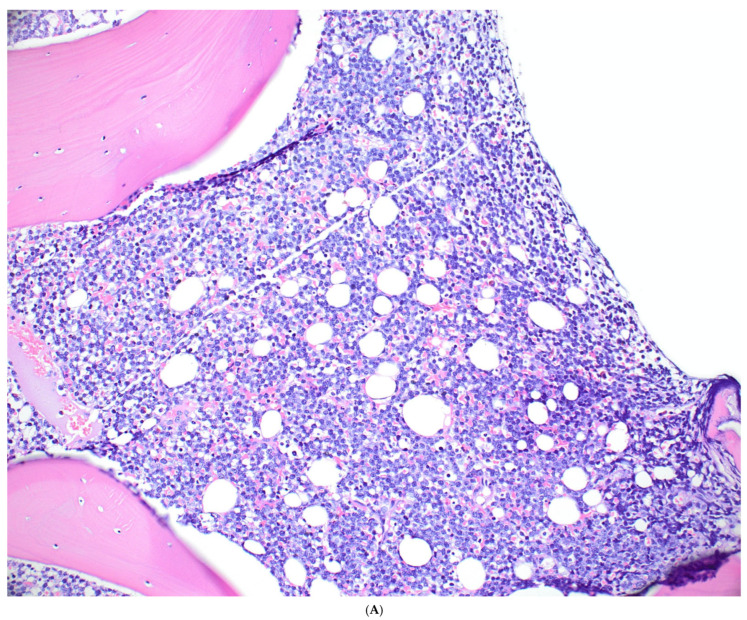

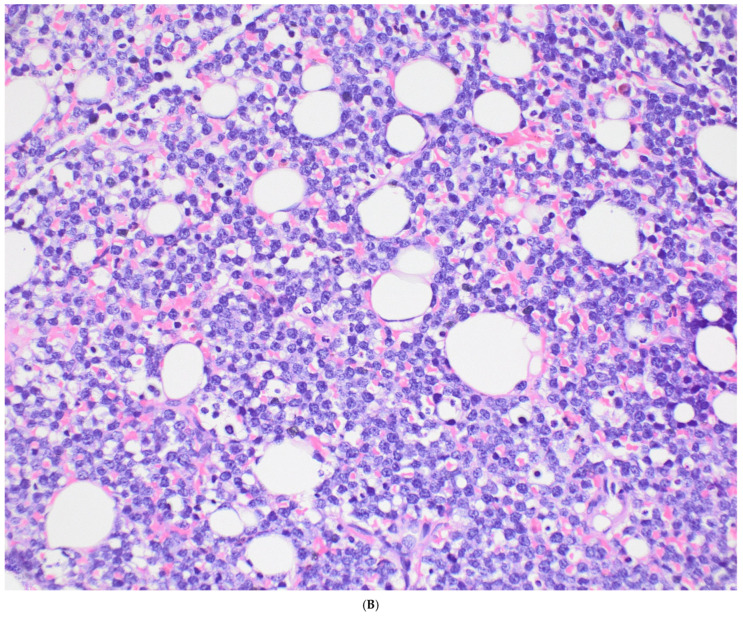

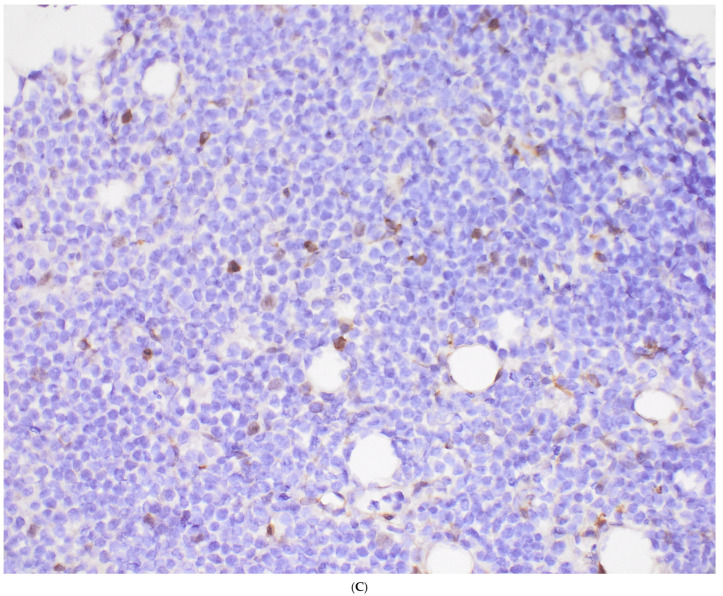

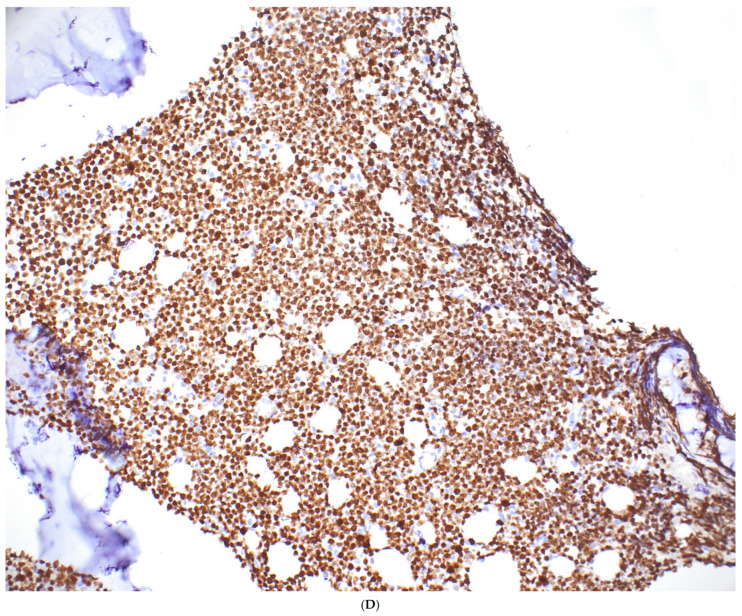
A representative case of BL involving the bone marrow. (**A**) The bone marrow is involved by BL in a diffuse pattern. (**B**) High-power view shows lymphoma cells of intermediate size with round to slightly irregular nuclei, dispersed chromatin, and several small and distinct nucleoli. Immunohistochemical analysis demonstrated that the lymphoma cells express B-cell-specific/characteristic antigens. BCL2 is negative (**C**), Ki67 (**D**) demonstrates a high proliferation rate approaching 100%.

The histologic features of BL are similar across epidemiologic variants. Tumor biopsy specimens are characterized by a diffuse and monomorphic population of predominantly medium-sized lymphoid cells with round nuclei, finely clumped and dispersed chromatin, multiple basophilic paracentrally located nucleoli, and basophilic cytoplasm. The tumor cells are arranged in sheets and there are many mitotic figures, as well as a high number of apoptotic bodies [126]. A “starry sky” pattern is usually present, resulting from the interspersed tingible body macrophages. Extensive necrosis is common. The histologic features of a typical BL case are shown in Figure 2. In bone marrow trephines, the neoplastic cells can be present with either a nodular or diffuse growth pattern and may not show the starry sky pattern, particularly in cases with less extensive infiltrates. A representative case of BL with bone marrow involvement is shown in Figure 3. Box 1 summarizes the diagnostic features of BL.

**Figure 4:**
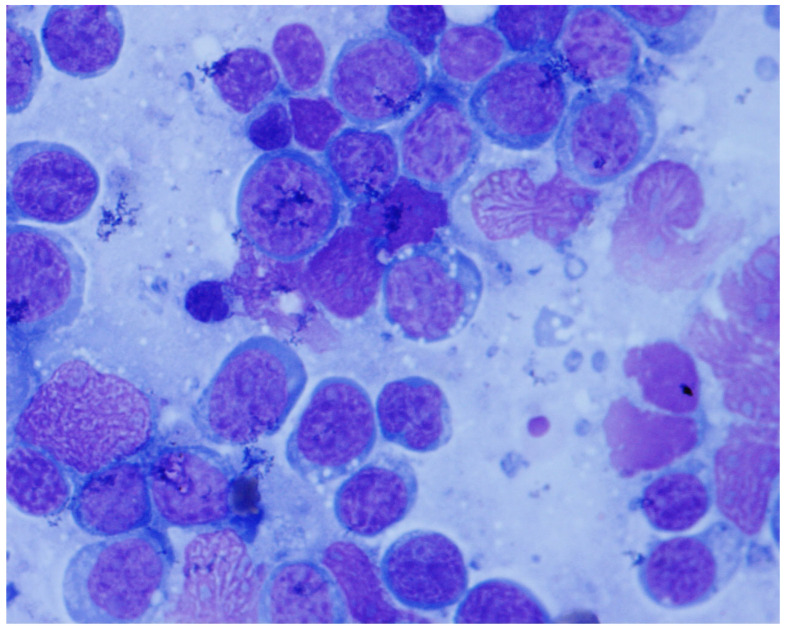
Cytologic smear collected by fine-needle aspiration from a lymph node with BL infiltrate. The neoplastic cells exhibit a basophilic cytoplasm containing many small lipid vacuoles.

On cytologic smears obtained by fine-needle aspiration, the lymphoma cells exhibit round to oval nuclei and a rim of basophilic cytoplasm containing many small lipid vacuoles, as shown in Figure 4. These lipid vacuoles are best visualized in air-dried specimens stained with neural fat stains such as oil red O. The background contains numerous apoptotic bodies and tingible body macrophages. Peripheral blood smears and bone marrow aspirate smears display similar cytologic features as those observed in fine-needle aspiration smears.

Several morphologic variants of BL have been described. A small subset of cases is associated with a florid granulomatous reaction that may mask the tumor [127,128]. Granai et al. reported that in these cases a proinflammatory response is triggered by Th1 lymphocytes and M1 polarized macrophages. This proinflammatory response may help maintain the tumor cells in a self-limited state or induce regression, which is associated with good prognosis [129]. In particular, ID-BL cases may show some plasmacytoid differentiation. Although BL is characterized in most cases by the above-mentioned monomorphic infiltrate of intermediate-size neoplastic cells, some cases can show more variable cell size and increased nuclear polymorphism with more prominent and fewer nucleoli. In the past, the term “atypical Burkitt lymphoma” has been used for such cases. The gene expression profile for these atypical cases has been found to be very similar to classic BL cases, making the term atypical BL obsolete (see below).

### 4.2. Immunophenotype

The characteristic immunophenotype of BL includes expression of all B-cell-characteristic antigens (CD19, CD20, CD79a, CD22 and PAX5) and many germinal center-associated antigens such as CD10, BCL6, HGAL and ME2FB, but LMO2 is often negative [130,131]. It has been reported that lack of LMO2 expression is associated with *MYC* translocation [132] and a combination of negative LMO2 and positive CD38 may aid in the diagnosis of BL [132,133]. Expression of the germinal center marker GCET is variable [130]. The neoplastic cells display additional features of mature germinal-center B cells including strong membrane IgM with light-chain restriction. In approx. 10% of patients, a sole IgA expression can be observed. There also exist rare cases with absence of surface immunoglobulins. BL cells are usually negative for CD3, CD5, CD23, CD30, CD138, CD44, terminal deoxynucleotidyl transferase (TdT), and BCL2. However, weak BCL2 expression can be observed in approx. 20% of patients and is still acceptable for BL diagnosis. A study showed no significant association between BCL2 positivity, clinical presentation and outcome [134]. SOX11 expression is detected in about 50% of EBV-negative cases and there is mutual exclusivity between SOX11 expression and EBV association [135]. IRF4/MUM1 is reported to be expressed in approximately 10–40% of the cases, ranging from occasional to most BL cells being positive, mostly with weak or variable intensity [136]. LEF1 is reported to be expressed in most BL cases [137]. MYC is moderately to strongly positive in the majority of neoplastic cells. Rare *MYC*-rearranged cases do not express MYC. It has been suggested that this might result from molecular alterations that induce a switch from *MYC* to *MYCN* [138]. In addition, MYC mutation may inhibit antibody binding. The Ki67 proliferative index is typically >95% (with monotonously intense staining), reflecting the high proliferation rate typical of this lymphoma. As EBV-associated BL exhibits a latency type 1, EBER in situ hybridization or immunohistochemical detection of EBNA1 are required to verify EBV presence, but not immunostains for LMP1. Cytoplasmic lipid vacuoles can be demonstrated employing an antibody against adipophilin [139].

### 4.3. Cytogenetic and Molecular Findings

The molecular hallmark of BL is the t(8;14) translocation of MYC from chromosome 8 to the immunoglobulin heavy-chain region on chromosome 14 in 70–80% of the cases or its variants; the t(2;8) translocation from chromosome 8 to the immunoglobulin kappa locus on chromosome 2 in 15% of the cases, or the t(8;22) translocation from chromosome 8 to the immunoglobulin lambda locus on chromosome 22, in 5% of the cases. *MYC* translocations occur in all BL variants but are also seen in other aggressive B-cell lymphomas. Assessment of *MYC* rearrangement is critical for the diagnostic workup of BL. Standard fluorescence in situ hybridization (FISH) using a break-apart probe is the preferred diagnostic method in most laboratories and can detect *MYC* rearrangements in approximately 90% of BL cases. It has been reported that an *IGH* and *MYC* fusion probe set can detect some cases that are missed using a break-apart probe [140,141]. Unbalanced *MYC* break-apart patterns with loss of the red or green signal frequently show *MYC* rearrangement detected by sequencing. Therefore, it is suggested that they should be interpreted as positive for *MYC* rearrangement in the appropriate clinical context [142,143]. Rare cases of BL have been shown to carry cryptic insertions of *MYC* into the immunoglobulin heavy chain [56].

A complex karyotype or multiple imbalances are uncommon at initial diagnosis of BL and should challenge the diagnosis, requiring additional testing. Mutations in genes controlling cell proliferation, growth and survival have been identified in BL, but there is no oncogene besides *MYC* that characterizes all BL cases. In 2006, two seminal publications regarding the molecular profiling of BL appeared simultaneously [56,144]. Both groups of authors took advantage of gene expression profiling methods (using Affymetrix chips) to try and define a specific expression signature of “*bona fide*” BL, which might distinguish it from other aggressive B-cell lymphomas, including diffuse large B-cell lymphoma (DLBCL), NOS, high-grade B-cell lymphoma (HGBCL), NOS and primary mediastinal B-cell lymphoma (PMLBCL). Although using somehow different experimental design and bioinformatical pipelines, both groups were able to define a robust gene expression signature for BL (the molecular Burkitt, or mBL, signature). It is very interesting to see how in these papers almost all cases classified as “atypical” BL showed a gene expression profile of mBL, hinting at the fact that the morphological spectrum of BL might be broader than previously accepted; both groups also detected cases that, despite showing a typical DLBCL morphology, were assigned with high probability to the mBL group, while HGBCL, NOS were, in most instances, molecularly classified as DLBCL. Among these cases, those that could be analyzed cytogenetically were either classified as “*MYC*-simple” (i.e., showing a rearrangement of *MYC* to either IG heavy- or light-chain gene segments, together with a low cytogenetic complexity) or “*MYC*-complex” (showing also rearrangements of *MYC* involving non-IG partners and a high genetic complexity), with the latter group showing a dismal prognosis [56]. These findings were later confirmed in a study focusing on the cytogenetic features of “*bona fide*” BL, atypical BL and “discrepant” BL (defined as cases of DLBCL with a mBL signature) [145], also showing that the latter category contained some “double-hit” lymphomas with translocations of both *BCL2* and *MYC* [145]. This latter category most probably has a large degree of similarity to the so-called “molecular high-grade” (MHG) group of DLBCL [146] and with the group of DLBCL showing a double-hit/dark zone signature (DH/DZ) [147], two largely overlapping signatures defining a group of aggressive B-cell lymphomas [148] which might benefit from tailored therapies [149]; these are, despite similarities at gene expression level, biologically distinct from “true” BL.

### 4.4. Diagnosis in Resource-Limited Parts of the World

As already presented, eBL has a significantly higher incidence in the resource-limited parts of the world. In these regions, clinical presentation plays a major factor, with many cases exhibiting tumors in characteristic locations such as the jaw and facial bones, breast, gonads, and gastrointestinal tract. In addition, fine-needle aspiration cytology is frequently used to establish diagnosis, and many patients are treated simply relying on clinical presentation and the presence of small round blue cells on fine-needle aspirate slides, without any possibility to perform tissue biopsy or IHC. Studies initiated by Lorenzo Leoncini and Kikeri Naresh highlighted the fact that 24% of cases diagnosed as BL with such an approach did not conform to BL upon review by an expert panel. Therefore, the authors and their co-workers designed an algorithm for the diagnosis of BL applicable for these settings [150] that has been amended in a more recent paper [151]. The proposed algorithm for BL diagnosis also contains strategies for alternative diagnoses that have to be considered in the differential diagnosis of BL. According to this algorithm, the diagnosis of BL requires the typical morphological features, a characteristic phenotype (expression of CD20, CD10, and CD38; negative or very weak BCL2 expression in 20% of the cases; lack of CD44 expression), intense MYC expression in ≥80% of cells, and a Ki67 index of >95%. Such cases do not need additional FISH studies in a setting in which FISH investigations are not available/feasible. If the criteria mentioned are not met, FISH studies for *MYC*, *BCL2* and *BCL6* (ideally along with IG genes) will have to be undertaken for further precise classification. Regarding cases with mild variations in cell, nuclear, and nucleolar size, and rarer cases exhibiting plasmacytoid features, the presence of *MYC* rearrangement only and a typical BL phenotype are compatible with a diagnosis of BL.

Box 1 **Diagnostic criteria for Burkitt lymphoma (according to WHO-HAEM5).**

*Essential*

• Medium-sized, monomorphic lymphoma cells with basophilic cytoplasm and multiple small nucleoli
• Expression of CD20 and CD10; absence or weak expression of BCL2; Ki67 index > 95%
• Usually strong expression of MYC (in >80% of cells) and/or demonstration of MYC breakage or IG::MYC translocation

*Desirable*

• Starry sky pattern, cohesive growth pattern
• Expression of BCL6 and of CD38, absent expression of terminal deoxynucleotidyl transferase (TdT)
• Absence of *BCL2* and *BCL6* translocations (mainly required in adult patients)

## 5. Differential Diagnosis (Key Points Summarized on Table 1)

### 5.1. High-Grade B-Cell Lymphoma with 11q Aberration

High-grade B-cell lymphoma with 11q aberration (HGBCL-11q) is an aggressive B-cell lymphoma characterized by a complex chromosomal 11q gain/loss-pattern. The tumor is rare and most cases have been diagnosed in children/adolescents and mainly in patients < 60 years of age.

**Figure 5:**
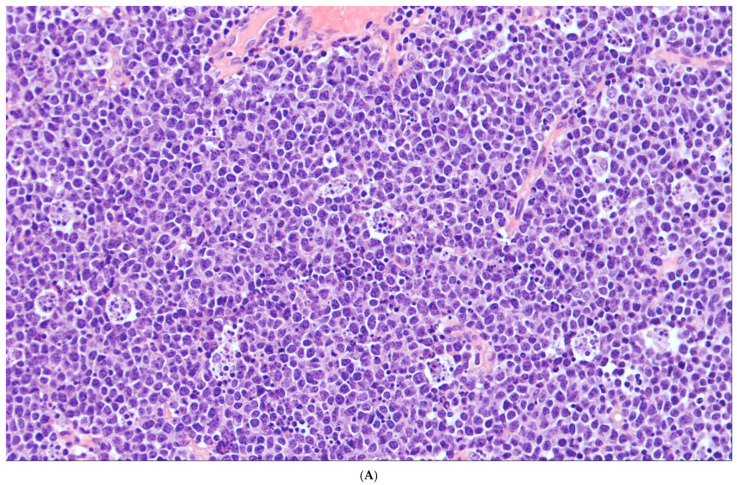

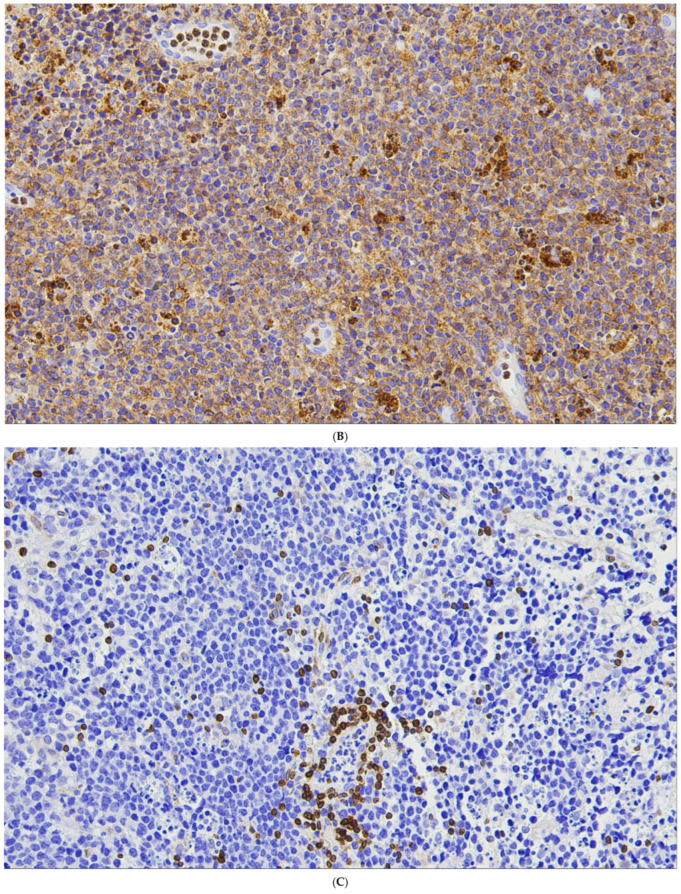

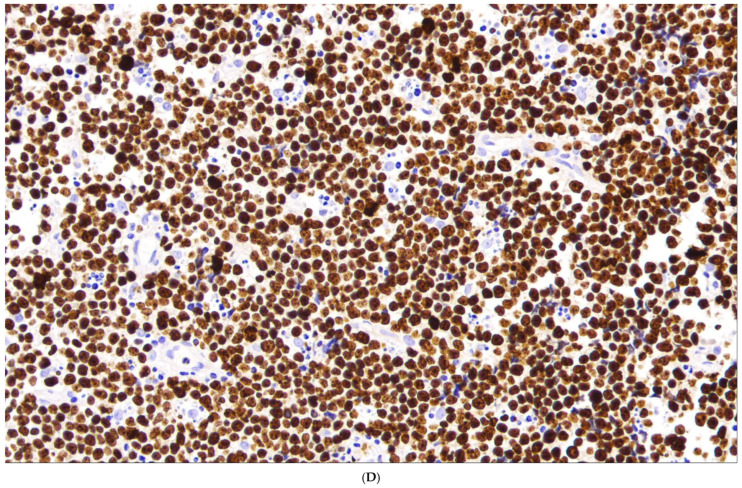
High-grade B-cell-lymphoma with 11q aberration. (**A**) There is a diffuse infiltration of medium-sized blastic cells slightly larger and more pleomorphic than those of Burkitt lymphoma with cohesive growth and a starry sky pattern with coarse apoptotic debris within the macrophages (H&E). (**B**) The tumor cells are strongly positive for CD10 and (**C**) negative for BCL2. (**D**) The Ki67 proliferation index is high.

HGBCL-11q enters the differential diagnosis with BL because of its architectural and cytomorphologic/immunophenotypical similarities to this lymphoma. The tumor cells are medium-sized with a small cytoplasmic rim and a coarse chromatin distribution similar to that of BL [152]. They are often arranged in diffuse cohesive sheets and show a starry sky pattern often with strikingly coarse apoptotic debris within the large macrophages [153]. Compared with BL, HGBCL-11q shows some variation in nuclear size and more prominent nuclei. More rarely, cases with frank large B-cell morphology have been described [154]. Next to its cytomorphological features, HGBCL-11q in almost all cases displays an identical immunophenotype as compared with BL: reactivity for CD10 and BCL6, negativity (or sometimes weak reactivity) for BCL2 and a high proliferation index (PI) of >90% (Figure 5) [155]. In flow cytometry, expression of CD16, CD56 and CD8 and lack of CD38-high expression have been described as typical features separating HGBCL-11q from BL [156]. The MYC protein is variably expressed and LMO2—in contrast with BL—is usually expressed.

The main difference with BL is—by definition—the virtual absence of a *MYC* translocation/rearrangement, although gene expression profiling has illustrated similarities between BL and HGLBCL-11q [152]. Instead, there is a complex aberration pattern involving the long arm of chromosome 11 (11q) with a minimal gained region in 11q23.3 and a minimal region of loss in 11q24.1-qter. Additional genetic differences with BL are illustrated by the different mutational landscape featuring mutations in genes such as *KMT2D*, *SWI/SMF*, *CREBBP* and *GNA13* and the virtual absence of Epstein–Barr virus (EBV) in HGBCL-11q [157]. Specific sets of overexpressed miRNAs in HGBCL-11q when compared to BL have been identified [158]. More recently, cases with dual rearrangements of *MYC* and an 11q aberration pattern have been described, which by definition are excluded from the entity [159]. Preliminary data indicate biological features intermediate between BL and HGBCL-11q or more similar to BL [160].

In practical terms, cases with a “like Burkitt” morphology and a matching immunophenotype, but that are negative for the *MYC* rearrangement, should undergo testing for the 11q aberration pattern [153]. This can be done by FISH or by next generation sequencing/array-based comparative-genomic hybridization. However, FISH may miss around 10% of cases with loss of heterozygosity at 11q.

### 5.2. High-Grade B-Cell Lymphoma with MYC and BCL2 or MYC and BCL6 Rearrangements (HGBCL-MYC/BCL2 and HGBCL-MYC/BCL6)

The entity of diffuse large B-cell lymphoma/High-grade B-cell lymphoma with *MYC* and *BCL2* rearrangements (with and without additional *BCL6* rearrangement (DLBCL/HGBCL-*MYC*/*BCL2*) is defined by structural chromosomal aberrations with breakpoints at both *MYC* and *BCL2* loci. According to the WHO-HAEM5 [155], these cases are differentiated by morphology with DLBCL-*MYC*/*BCL2* diagnosed in cases with large-cell morphology and HGBCL-*MYC*/*BCL2* diagnosed in cases with high-grade morphology. DLBCL/HGBCL-*MYC*/*BCL2* usually has a germinal center B-cell-like (GCB-like) immunophenotype with a high number of cases positive for CD10 and BCL6 and varying reactivity for IRF4/MUM1 [161]. By definition, there is an *MYC* rearrangement and MYC protein is expressed in a high number of nuclei. The Ki67-PI is more variable than in BL; however, cases with PIs of >90% do exist. HGBCL-*MYC*/*BCL2* show a diffuse growth pattern in the majority of cases and have either an intermediate morphology between DLBCL and BL, or a blastoid morphology; however, this latter distinction seems not to be well reproducible [162] (Kurz et al. submitted). The pathogenesis of DLBCL/HGBCL-*MYC*/*BCL2* has a close relationship to follicular lymphoma and GCB-type DLBCL. *MYC* rearrangements have distinct architectural features [163]. Its mutational spectrum encompasses genes frequently mutated in follicular lymphoma such as *BCL2*, *KMT2D*, *CREBBP*, *EZH2* and *TNFRSF14*, next to genes commonly mutated in GCB-type DLBCL or Burkitt lymphoma such as *CCND3*, *SMARCA4* or *MYC* itself [164]. Two distinct gene expression signatures have been described in DLBCL/HGBCL-*MYC*/*BCL2* termed “double hit” and “molecular high grade” [146,147] that detect “dark zone” biology and hence, were renamed “dark zone” signature [165].

In contrast, DLBCL/HGBCL-*MYC*/*BCL6* clearly shows more heterogeneous biological features with variable gene expression profiles and mutational spectrum [166]. In WHO-HAEM5, they are listed among DLBCL, NOS or HGBCL, NOS, depending on morphology.

**Figure 6:**
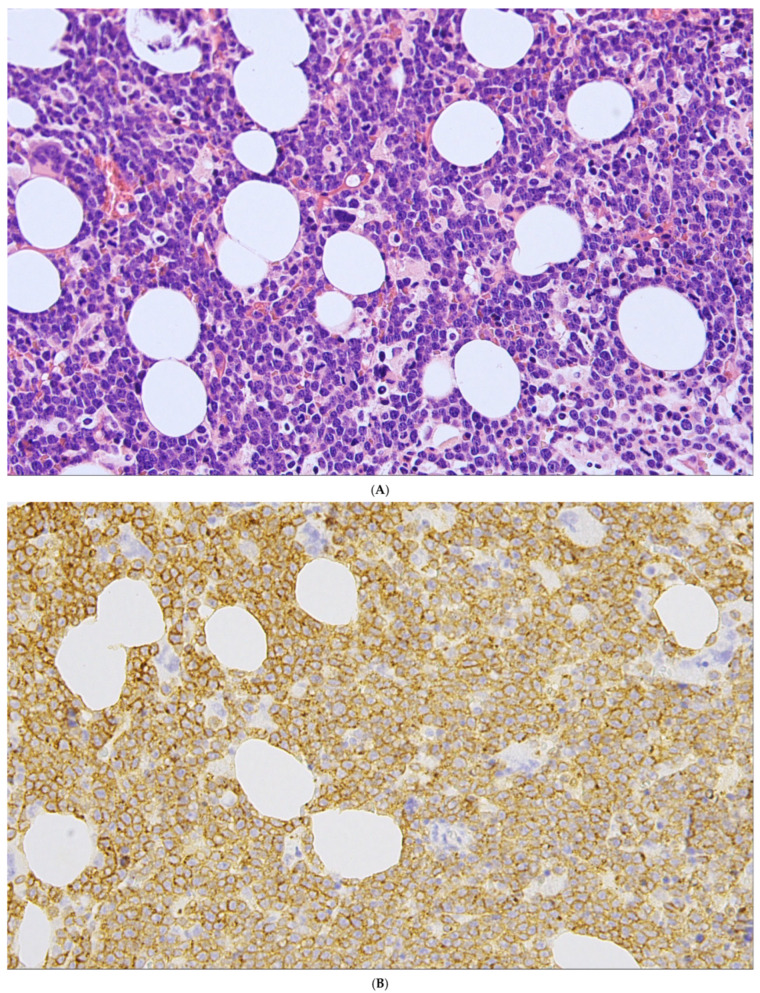

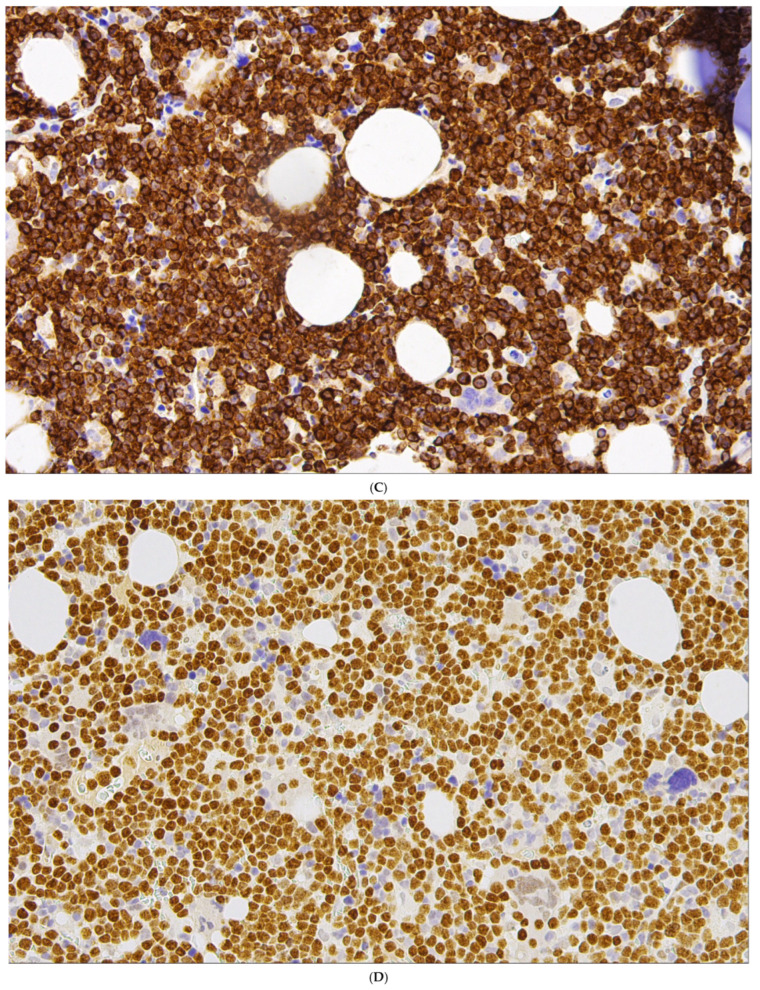
High-grade B-cell lymphoma with *MYC* and *BCL2*-rearrangements. (**A**) This example of HGBCL-MYC/BCL2 shows “like-Burkitt” features with medium-sized tumor cells that have cohesive growth. There is also a prominent starry sky pattern. (**B**) The tumor cells strongly express CD10, but in contrast to Burkitt lymphoma, BCL2 (**C**) is also strongly positive. (**D**) The proliferation index is high but not as high as in Burkitt lymphoma.

HGBCL-*MYC*/*BCL2* or *MYC*/*BCL6* may look similar or even identical (Figure 6) to BL. However, the vast majority of HGBCL-*MYC*/*BCL2* do (strongly) express BCL2 in contrast to BL, whereas BCL2 expression in HGBCL-*MYC*/*BCL6* is more variable. The finding of a *BCL2* or a *BCL6* break, or both, in a *MYC*-rearranged “like-Burkitt” lymphoma by definition excludes BL. Therefore, in cases with high-grade or like-Burkitt morphology, and especially in those with aberrant morphological features, after identification of a *MYC* rearrangement, additional testing for *BCL2* and/or *BCL6* rearrangements is warranted.

### 5.3. High-Grade B-Cell-Lymphoma (HGBCL) NOS

**Figure 7:**
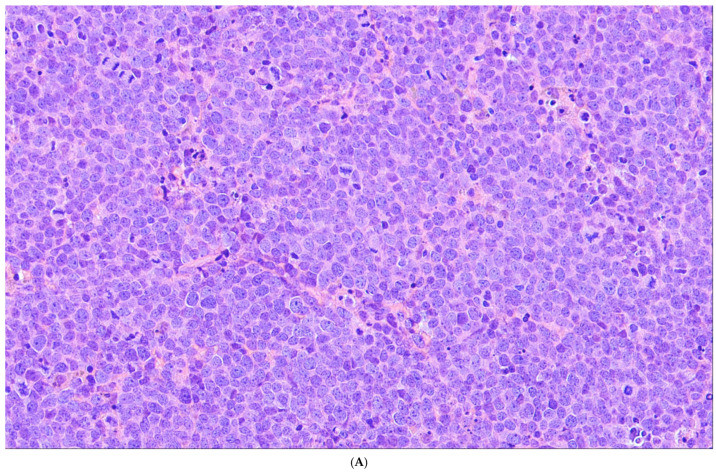

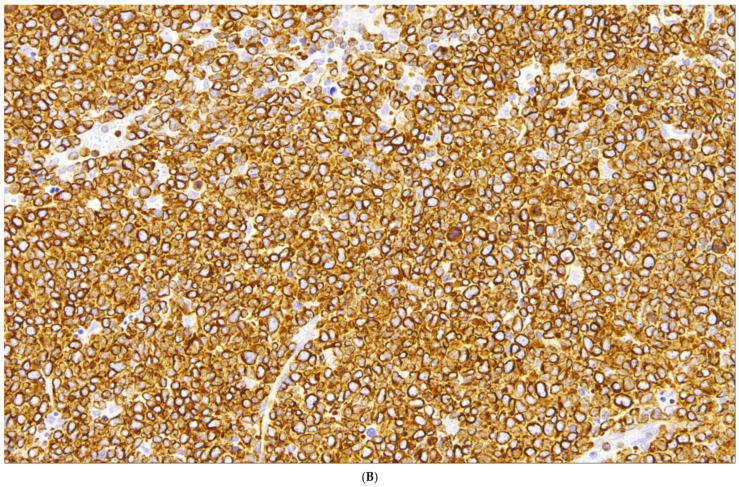
High-grade B-cell-lymphoma NOS. (**A**) This tumor shows a morphology intermediate between DLBCL and BL with a diffuse proliferation of predominantly medium-sized cells exhibiting moderate cohesive growth and round, homogeneous nuclei with finely dispersed chromatin. (**B**). In contrast with Burkitt lymphoma, the tumor cells strongly express BCL2. No rearrangements of *MYC*, *BCL2* or *BCL6* were detected. The complex 11q gain/loss pattern typical of HGBCL-11q was absent.

High-grade B-cell-lymphoma (HGBCL) NOS is a heterogeneous category of aggressive mature B-cell lymphomas composed of medium-sized or blastoid cells that do not form part of other defined lymphoma entities [155]. It usually shows a diffuse proliferation of intermediate or blastoid cells with variable cytoplasmic features and a more pronounced nuclear pleomorphism than what is usually encountered in BL. A starry sky pattern may occasionally be seen. HGBCL NOS expresses—next to pan B-cell markers—a germinal center B-cell-like immunophenotype in the majority of cases (Figure 7) [162,167]. In a recent publication from the Lymphoma/Leukemia Molecular Profiling Project (LLMPP), CD10 was positive in 74% and BCL2-expression was recorded in 57% of cases. However, the inter-observer agreement regarding the diagnosis was low and in the LLMPP paper, less than half of the submitted cases were finally accepted by the panel (Kurz et al. submitted).

The genetic landscape of HGBCL NOS is heterogeneous. In the recent report from the LLMPP, 59% were germinal center B-cell-like (GCB), and 25% were activated B-cell-like (ABC) by cell-of-origin. The LymphGen genetic classifier for DLBCL-NOS assigned a genetic subtype to 34% of HGBCL NOS. *MYC* rearrangements and dark zone signature expression were found in 47% and 45% of cases, respectively, clearly surpassing numbers in DLBCL-NOS. There were also frequent mutations in *ID3*, *MYC*, *CCND3,* and *TP53* genes.

Since single *MYC* rearrangements (without accompanying *BCL2* and *BCL6* breaks) frequently occur in HGBCL NOS; this entity forms a valid differential diagnosis to Burkitt lymphoma, especially also taking into account that classic FISH using break-apart or fusion probes may miss *MYC* translocations in around 10% of cases [168]. In lymphomas without a double hit and without *MYC* translocations but featuring high-grade morphology, absence of the 11q gain/loss pattern of HGBCL-11q should be confirmed.

### 5.4. Diffuse Large B-Cell Lymphoma

Next to HGBCL NOS, DLBCL forms the most important differential diagnosis with BL. Diffuse large B-cell lymphoma has a broad spectrum of architectural features, including cohesive growth and a starry sky pattern in some cases, and of cell sizes including “small-cell” variants of centroblasts and/or immunoblasts. It can be CD10-positive and BCL2-negative and may have a high proliferation index. In addition, the *MYC* translocation is found in 6–10% of the cases [169,170], and in 1/3 of these, *MYC*R occurs as the single translocation (without additional *BCL2* and *BCL6* rearrangements).

**Figure 8:**
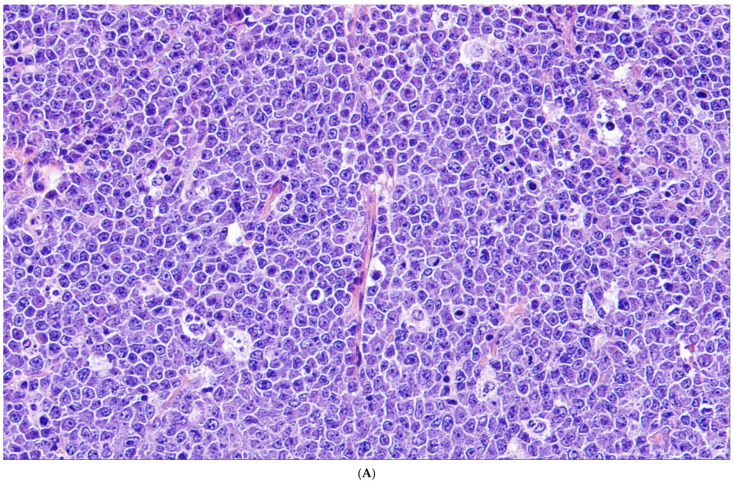

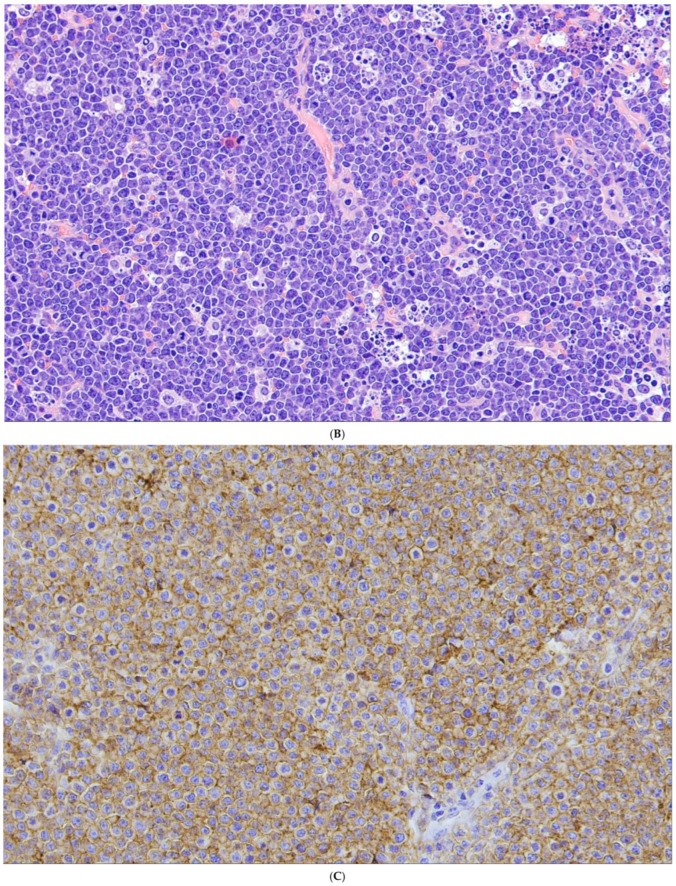

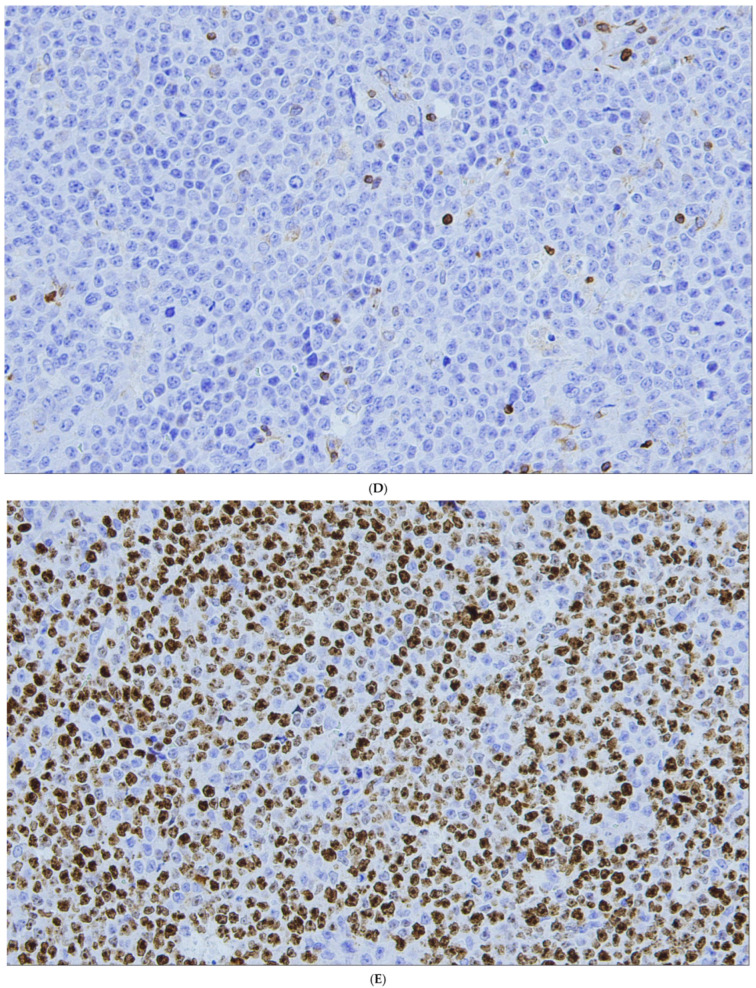

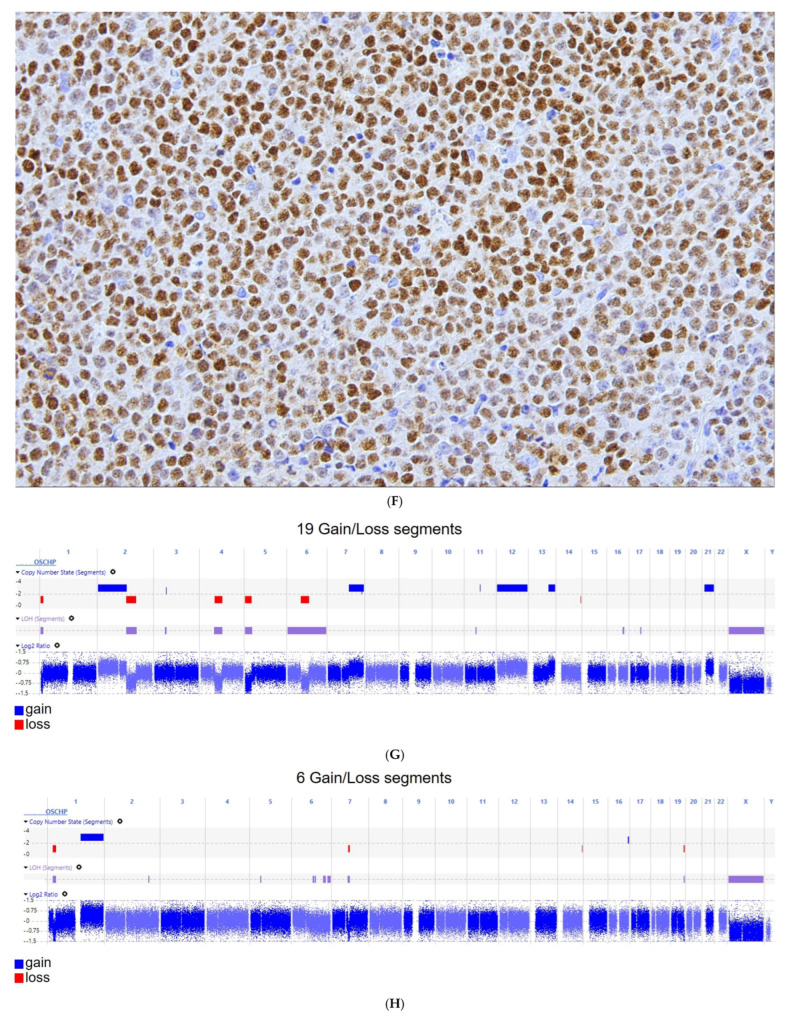
Diffuse large B-cell lymphoma. (**A**): In some cases, DLBCL may show a cohesive growth pattern and be characterized by a starry sky pattern, but usually harbors larger cells then those in Burkitt lymphoma (**B**). (**C**): In this case, the DLBCL was positive for CD10. BCL2 was negative or only weakly expressed (**D**) The proliferation index was high (**E**). LMO2 was positive in the majority of tumor cells (**F**). After identification of a *MYC* rearrangement, additional molecular analyses were initiated. NGS-analysis disclosed pathogenic variants in *XPO1*, *IRF8*, *CREBBP* and *MYC* and a complex karyotype in Oncoscan analysis (**G**), whereas the Burkitt lymphoma in B exhibited mutations in *ID3* and *MYC* and low genetic complexity in Oncoscan analysis (**H**). Finally in the DLBCL tumor in A, an additional *BCL2* translocation was also identified finally leading to the diagnosis of DLBCL-*MYC/BCL2*. The protein negativity for BCL2 suggests a mutation involving the binding site of the antibody.

However, the overall coincidence of immunophenotypic and genetic features characterizing BL (CD10 and BCL6 positivity, absence (or, rarely, weak expression) of BCL2, Ki67-index > 95%, strong expression of MYC, and presence of *MYC* breakage or IG::*MYC* translocation) is rare in DLBCL [171], occurring in <1% of cases. In diagnostically difficult cases, more sophisticated molecular testing can be performed; however, mutation profiling may not be the optimal method, since mutations in “Burkitt-typical” genes such as in *ID3*, *CCND3*, *GNA13*, *SMARCA4* and *MYC* itself have also been found altered in DLBCL or HGBCL NOS (as well as in DLBCL/HGBCL-MYC/BCL2) [172]. Array-based comparative genomic hybridization and/or whole genome sequencing may be an alternative, indicating a low frequency of overall chromosomal imbalances in BL compared with DLBCL (Figure 8). In equivocal cases, methylation profiling may be of help having identified specific methylation patterns in BL cells [119,173,174].

### 5.5. B-Lymphoblastic Leukemia/Lymphoma

B-lymphoblastic leukemia/lymphoma (B-ALL/LBL) represents a neoplasm of precursor lymphoid cells of B-cell lineage involving the bone marrow and also often extramedullary, nodal or extranodal sites.

Morphologically, there is a diffuse and sometimes cohesive proliferation of medium-sized blasts with a uniform appearance. They have a scarce cytoplasm, round to oval nuclei, and a finely dispersed nuclear chromatin. Sometimes, a starry sky pattern can be seen. On immunohistochemistry, next to B-cell markers and especially CD79A and PAX5, B-ALL/LBL can express CD10 and may also be negative or weakly positive for BCL2. In many cases, CD34 and TdT are expressed; therefore, in questionable cases, staining for these two markers is recommended, the expression of which usually proves precursor cell disease. However, CD34 expression can be absent, and TdT can also be expressed in non-precursor B-cell lymphomas [175]. In addition, rare cases of B-ALL/LBL may harbor rearrangements of *MYC* or *MYC* and *BCL2* [54], sometimes creating a challenging differential diagnosis, e.g., in cases of HGBCL-MYC/BCL2 with TdT expression [175].

**Table 1:** Differential diagnosis of aggressive B-cell lymphomas.

Entity	Morphology	Immunophenotype	Cytogenetic Findings
Burkitt lymphoma (BL)	Cohesive monomorphic, medium-sized cells with multiple small nucleoli and basophilic cytoplasm	B cells positive for CD20, CD10 and MYC protein, Ki67 > 95%, absent or weak expression of BCL2, negative for TdT and LMO2	*MYC* translocation, most often IGH::*MYC*, absence of *BCL2* or *BCL6* translocations
High-grade B-cell lymphoma with 11q aberration	Morphology resembling BL but more pleomorphism with some variation in nuclear shape/size and presence of larger nucleoli and coarse debris in starry sky macrophages in some cases	Identical immunophenotype to BL, variable MYC expression	Minimal gain in 11q23.3 and a minimal region of loss in 11q24.1-qter, lack of *MYC* translocation
High-grade B-cell lymphoma with *MYC* and *BCL2* or *MYC* and *BCL6* rearrangements	Variable morphology large-cell or blastoid/high-grade morphology	Germinal center B-cell-like positive for CD10 and BCL6 and varying reactivity for IRF4/MUM1, Ki67 more variable than BL, most (with *MYC* and *BCL2* translocation) strongly express BCL2, in occasional cases subset of cells expressing TdT	*BCL2* translocation in addition to *MYC* translocation/ *BCL6* translocation in addition to *MYC* translocation
High-grade B-cell lymphoma, NOS	Medium-sized or blastoid cells, variation in nuclear size and nucleolar content; cohesive growth usually absent	Germinal center B-cell-like in the majority of the cases, most express strongly BCL2	Single *MYC* rearrangements in a proportion of cases (without accompanying *BCL2* and *BCL6* breaks)
Diffuse large B-cell lymphoma (DLBCL)	Broad spectrum of architectural features including cohesive growth and variable cell size	Can be CD10-positive and BCL2-negative and may have a high proliferation index	*MYC* translocation is found in 6–10% of the cases and in 1/3 of these *MYC* rearrangement occurs as single translocation
B-lymphoblastic leukemia/lymphoma	Medium-sized blasts with uniform appearance. Fine nuclear chromatin; nucleoli often less conspicuous	Uniform expression of TdT in most cases; CD34 expression in a subset of cases. Frequently reduced expression of CD20 and weakly positive for BCL2. CD10 positive in a subset	Rarely *MYC* single translocations or *MYC* and *BCL2* dual translocations presence of other key defining rearrangements/translocations

## 6. Outlook/Conclusions

The diagnosis of BL is apparently straightforward from a pathological point of view, but the differential diagnosis with DLBCL can be morphologically difficult and prone to low inter-observer reproducibility, especially in the era of needle biopsies. This distinction is not trivial, since BL patients require substantially different therapeutic regimens, which might have severe toxic consequences, especially in adult patients, while CHOP-like regimens commonly used in DLBCL are not optimal for BL [176]. In consideration of the decreasing role of morphological analysis in the era of small (and sometimes crushed) biopsies, a wider application of mutation analysis and possibly of gene expression or methylome profiling to achieve a reproducible diagnosis of “biologically true” BL is therefore a desirable goal of hematopathology for the near future. A future deeper understanding of the biology of BL through the use of adequate experimental models might also detect novel exploitable molecular targets, such as the case of BCL6 [177], although the prospect of “entity agnostic” therapies such as bi-specific antibodies might revolutionize the approach to lymphomas, including BL, in the upcoming years.
